# Reverse microdialysis of sucrose stimulates soil fungal and bacterial growth at the microscale

**DOI:** 10.1186/s12866-025-04082-5

**Published:** 2025-07-14

**Authors:** Andreas N. Schneider, Scott Buckley, Zulema Carracedo Lorenzo, Regina Gratz, Lina Nilsson, Mark Swaine, Nathaniel R. Street, Andy F. S. Taylor, Sandra Jämtgård

**Affiliations:** 1 Department of Plant Physiology, Umeå Plant Science Centre, Umeå University, Umeå, SE-901 83 Sweden; 2 Department of Forest Ecology and Management, Swedish University of Agricultural Sciences, Umeå, SE-901 83 Sweden; 3 Department of Forest Genetics and Plant Physiology, Umeå Plant Science Centre, Swedish University of Agricultural Sciences, SE-901 83 Umeå, Sweden; 4 Science for Life Laboratory, Umeå University, Umeå, SE-901 83 Sweden; 5 The James Hutton Institute, Craigiebuckler, Aberdeen, AB15 8QH UK; 6 Institute of Biological and Environmental Sciences, Cruickshank Building, University of Aberdeen, Aberdeen, AB24 3UU UK

**Keywords:** Root exudation, Microdialysis, Fungi, Bacteria, Amplicon sequencing

## Abstract

**Background:**

The rhizosphere is a critical microenvironment that plays key roles in plant nutrient availability, largely due to root interactions with rhizospheric microbes. However, we lack suitable methods that can elucidate mechanisms determining rhizospheric community structure and function within the context of a dynamic, undisturbed soil. Microdialysis has been used for low intrusive soil nutrient sampling at the scale of a fine root, with small probes that also enable release of defined compounds. We evaluated whether microdialysis could simulate exudation, by the release of sucrose, and stimulate changes in a soil microbial community, allowing us to determine the microbes that responded most to carbon release.

**Results:**

Microdialysis successfully stimulated growth on probe surfaces of fungi and bacteria, which were extracted and sequenced for identification. Microbial growth was also visualized with scanning electron microscopy. The majority of the species stimulated were classified as fast growing or opportunistic, e.g. yeasts, moulds, proteobacteria and actinobacteriota, which are known to respond quickly (within days) to the release of simple sugars as exudates in the rhizosphere.

**Conclusions:**

The study demonstrates the potential of using microdialysis as a tool to investigate interactions between root exudation and soil microbial community composition, initially for individual compounds and in the future for more complex compositions.

**Supplementary Information:**

The online version contains supplementary material available at 10.1186/s12866-025-04082-5.

## Background

Soil microorganisms are key components of both natural and managed ecosystems. The heterogeneity of both soil structure and microbial communities in soils is huge, with microenvironments separated by only micrometres to millimetres making up distinct niches with differences in e.g. moisture, pH, oxygen, nutrients and substrate availability. This microspatial heterogeneity is exemplified by abrupt biotic and abiotic changes at the root-soil interface – the rhizosphere, where microbial densities are higher and diversity lower than in bulk soil [1–3]. This is a consequence of that plant roots releasing an array of exudates (e.g. energy and signalling molecules) [4], thus creating a unique, nutrient-rich niche where certain microbes are stimulated. There is currently considerable research on the rhizospheric microbiome, providing critical information on the presence and abundance of soil organisms [5, 6], with metagenomic and metabarcoding analyses having greatly expanded our understanding of the complexity of soil biodiversity, which was previously largely limited to culture-dependent studies [7–9]. In the absence of mycorrhizal fungi, the rhizosphere is generally viewed to extend to soil volumes up to 5 mm away from the root [10], and to further elucidate microbial recruitment the position and scale of sampling is of critical importance in pinpointing the function of rhizosphere communities in different soil environments [11].

Several inventive experimental set ups have been used in attempts to mimic root exudation and measure the effects on soil chemistry. Root exudation solutions can be supplied directly to the soil in a solution or via microlysimeters [12, 13]. Both these methods supply the substrate to the soil via mass flow, which differs from plant root exudation since applied solutions and lysimeters also release water in addition to the substrates. In contrast, root exudates move via diffusion, which could potentially affect the microbial community compositions and soil chemistry differently. As a complicating factor, plant root tips are usually surrounded by an exuded mucilage layer, which can attenuate plant stress [14] and act as carbon source and drought protection for microbes in the rhizosphere [15].

Another alternative to study the direct effects of exudates on microbiomes can be to use plant molecular tools like knock-out mutants and over-expressors of responsible plasma membrane transporters. The mechanisms of root exudation are still debated even though our understanding of mapping exudation under different settings, the involvement of root morphology and identifying physiological mechanisms – e.g. cell membrane transporters [16] is increasing. Currently, relatively few transporters have been identified compared to the numerous compounds identified in root exudates [16, 17], which hinders the generation of mutants for many known exudates. Difficulties in studying the roles of root exudates may also depend on whether their exudation requires active or passive transport. Bioactive secondary compounds (e.g. coumarin) seem to be actively exuded from roots through energy-consuming primary or secondary active transporters [17, 18]. Primary metabolites (mainly sugars, amino acids and organic acids), on the other hand, may be passively lost from the root at the meristematic root apex *via* diffusion [17, 18]. If that is the case, investigating this with different plant mutant lines might be more challenging.

As a complement to a plant molecular toolbox to explore mechanisms in root exudation, an alternative approach would be to use methods that can simulate the release of targeted compounds, e.g. root exudates, while simultaneously allowing sampling of the chemical response in the soil (e.g. nutrient availability) and enabling sampling of the responsive soil microbiome. Microdialysis can offer these possibilities and has recently been applied to measurine nitrogen and phosphorus availability at the root scale with minimal disturbance in soil [19, 20]. In addition to sampling the soil solution via induced diffusion, the microdialysis probe can simultaneously release compounds *via* diffusion [20–22]. In practice, this means the technique can be used to simulate release of root exudates and simultaneously sample nutrient availability. Previous work has shown that applying this simulation of exudation in soil can have effects on availability of phosphorus [22, 23] and nitrogen [20], and that the release of organic acids (using microdialysis) leads to immediate and dynamic changes in microbial growth, activity, and nitrogen mineralization in the surrounding soil [24]. This approach offers an opportunity for determining how plant root exudates affect rhizosphere microbiome community structure.

**Fig. 1:**
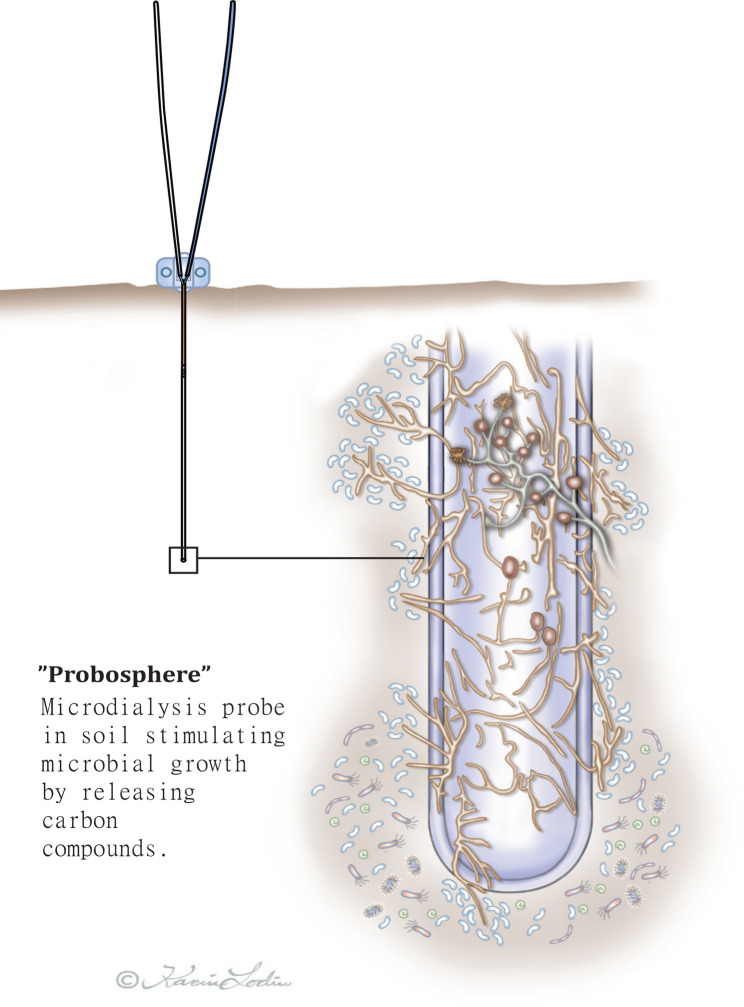
The ‘Probosphere’, an analogue to the rhizosphere. The diameter of the probe is 0.5 mm. The permeable probe (20 kDa molecular weight cut-off) is connected to a pump with two tubes (pictured above the soil line), and sucrose is pumped through the probe and released through the probe surface via diffusion. Illustration: Karin Lodin, Lodin designbyrå AB

Here, we built upon our previous work [20, 25, 26] by testing whether release of sucrose from a microdialysis probe stimulated microbial growth on the probe. To provide a proof of concept, we released a carbon source (in the form of sucrose) over a period of four days, using semipermeable microdialysis probes inserted in homogenised organic soil from a boreal forest. Since the composition of plant root exudates varies widely [16, 18, 27], we chose sucrose as a simplified starting model exudate, known to be exuded from living root tips [28]. As boreal forest soils are often nitrogen limited, we also added a nitrogen rich plant litter to some microcosms to artificially increase total nitrogen and potentially microbial activity. Scanning electron microscopy was used to visualize the microbes growing on the microdialysis probe surfaces and amplicon sequencing of fungal ITS and bacterial 16S rRNA regions was used for microbial community profiling. The aim of this study was to create a method that future studies could use for dissecting the roles of specific root exudates in rhizospheric microbial colonization, by creating a stimulated ‘probosphere’ (Fig. 1).

## Methods

### Soil sampling

Soil was collected in September 2019 from the organic horizon (0–5 cm) of a Scots pine forest with an understorey of ericaceous shrubs at the Rosinedal Research area, near Umeå, Sweden (64°10’20’’N,19°44’30’’E). The site soil consists of sand, with a weakly developed podzol and an organic layer ranging from 2 to 5 cm thickness, see Lim et al. [29] for a detailed description of the site. The annual mean precipitation is 587 mm, and the annual mean air temperature is 1.9 °C. Soil was taken from the non-fertilized control site and transferred to the laboratory for further processing. It was then sieved (2 mm mesh), mixed, and stored at 4 °C until use (4 months, January 2020). The soil contained 1.04 ± 0.03% N, 38.67 ± 1.07% carbon (carbon/nitrogen = 37.2), with a pH_H2O_ of 4.5.

### Microdialysis sampling

Seven g of soil, at 70% water holding capacity (3 g soil dry weight) without or with 30 mg litter (pea litter, C/N 14.8) added, was placed in 5 ml Eppendorf tubes (without lids) and left at room temperature (21 °C) to acclimatise for one week. Pea litter (from dried and milled *Pisum sativum*) was chosen to artificially increase nitrogen availability in the microcosm, as previously shown [20]. One microdialysis probe (30 mm x 0.5 mm, surface area 0.4732 cm^2^) with a 20 kDa molecular weight cut-off, (CMA 20; CMA Microdialysis AB, Solna, Sweden) composed of polyarylethersulphone (PAES) was inserted into the soil in each of the tubes after carefully making a hole in the soil with a guiding needle. Each probe was connected to a syringe (3 mL; BD Plastipak) on a syringe pump (CMA 4004 infusion pump) and perfused with either high-purity de-ionised water (MQ, from here on referred to as control samples) or sucrose (5 mM) at a flow rate of 1 µL min^−1^; for 16 h per day for a total of 4 days. Based on effluxes we have previously measured in Buckley et al. [20], this should amount to a range 80–250 µg of carbon released per probe over 4 days. There were 40 tubes in total (20 probes without litter and 20 probes with litter) for each perfusate type. As soon as pumping finished on the last day, the microdialysis probes were carefully removed from the soil. The microdialysis membrane was cut and peeled off the shaft of the microdialysis probe, with a sterile scalpel, into a sterile 1.5 ml Eppendorf tube and flash frozen in liquid nitrogen and stored at −80 ^o^C until DNA extraction. Four membranes were pooled to one extraction tube resulting in *n* = 5 for each of the four treatments: without litter (control and sucrose) and with litter (control and sucrose). Two replicates of the control without litter treatment and one replicate of the sucrose without litter treatment were accidentally destroyed during sample processing leading to *n* = 3 and 4 for these treatments.

### gDNA preparation

The frozen microdialysis membranes were homogenized six times for 1 min at max speed, using a Retsch bead mill, with each extraction tube containing two 3 mm diameter metal beads (washed in RBS- and MQ H_2_O and autoclaved). Samples were kept frozen using liquid nitrogen during the process.

The DNA on the microdialysis membranes was extracted using the FastDNA Spin Kit MP (MP Biomedicals, Irvine, CA, USA), with a modified protocol. 1 ml of CLS-Y buffer was added to the samples to prevent DNA degradation and keep the temperature low. The samples were then homogenised at max speed for 3 min, in the same Retsch bead mill. Thereafter the samples were centrifuged for 10 min at 14,000 rpm. 900 µl of the supernatant was transferred to a new Eppendorf tube and 800 µl Binding Matrix was added. The tube was inverted gently for 5 min at room temperature. Half of the suspension was added to a SPIN filter unit and centrifuged at 14,000 rpm for 1 min, when the liquid in the catch tube was discarded. The centrifugation was repeated with the remaining sample, and the catch tube was discarded. 500 µl SEWS-M buffer was added to the filter. The pellet in the filter unit was carefully resuspended, and then centrifuged again for 1 min at 14,000 rpm. The catch tube was discarded and replaced with a new Eppendorf tube. The filter was centrifuged for 2 min at 14,000 rpm to dry the pellet. The catch tube was once again discarded and replaced with a new Eppendorf tube. The DNA was eluted by resuspending the pellet in the filter unit with 80 µl DES. The tubes were capped and incubated at 55 °C in a heat block for 5 min. The filters were then centrifuged at 14,000 rpm for 2 min to elute the DNA in the new Eppendorf tube. DNA concentrations in the samples were measured using the Qubit dsDNA High Sensitivity (HS) Assay kit (Invitrogen, Carlsbad, California, US) with Qubit fluorometric quantitation (Invitrogen). The extracted DNA samples were stored at −80 °C until library preparation.

### Library preparation and sequencing

#### ITS region

For amplifying the ITS2 region, we used the primer pair gITS7 (GTGARTCATCGARTCTTTG; [30]) and ITS4 (TCCTCCGCTTATTGATATGC; [31]) containing Illumina adaptor overhangs (Table S1, Additional File 1).

0.5 ng sample DNA was amplified in triplicates using HotStar HiFidelity Polymerase Kit (Qiagen, Hilden, Germany). A custom fungal mock community described in Haas et al. [32] served as positive control and was treated the same as the experimental samples and negative water controls. PCR thermal cycling conditions were: initial denaturation at 95 °C for 5 min, followed by 35 cycles at 94 °C for 15 s, 55 °C for 1 min and 72 °C for 45 s. The final cycle was: 72 °C for 10 min and ending at 12 °C. After the PCR amplification, the triplicate products were pooled and subsequently purified from remaining PCR reagents using AMPure XP beads (Beckman Coulter, Indianapolis, USA). 20 µl of amplified product was mixed with 16 ul XP beads and cleaned using 80% ethanol according to manufacturer’s protocol and a magnetic 96-well plate (Thermo Fisher Scientific, Waltham, MA USA). After clean-up, samples were resuspended in 43 µl of 10 mM Tris (pH 8.5). In order to anneal unique barcode combinations per sample, this cleaned PCR product was further amplified using the same PCR cycling conditions and reagents as used in the previous PCR reaction step and 20 cycles, with index primers (Table S1). After the PCR, sample DNA concentration was again measured by Qubit. 5 µl of each sample PCR product was pooled together and mixed well. Subsequently, 100 µl of pooled PCR product was aliquoted into an Eppendorf tube. 150 µl of AMPure beads were added to the pooled library sample, and the pooled library was cleaned as described previously, but using 70% ethanol. Cleaned sample was resuspended in 250 µl of 10 mM Tris (pH 8.5). The concentration of the pooled library sample was measured by Qubit. To remove remaining contaminants and to select DNA fragments of the right size, the pooled library was cleaned using BluePippin according to the manufacturer’s protocol. We selected DNA fragments with sizes 350 to 800 bp, to account for variation in the length of the ITS region. Samples were diluted to 10 nM and sent to SciLifeLab (Stockholm, Sweden) for paired-end sequencing using Illumina MiSeq (Illumina, San Diego, CA), yielding 300bp forward and reverse reads.

#### 16S rRNA gene

For amplification of the 16S rRNA gene hypervariable V5-V7 region the primer pair 799 F (5′-AACMGGATTAGATACCCKG-3′) and 1193R (5´-ACGTCATCCCCACCTTCC-3´) [33] was used (Table S2, Additional File 1).

0.5 ng of sample DNA was amplified in triplicates using KAPA Hifi HotStart Readymix (Roche, Basel, Switzerland). The Zymobiomics microbial community DNA standard (Zymo research, Irivine, CA, USA) served as a mock community and was treated the same way as the samples and negative water controls. The PCR thermal cycling conditions were: initial denaturation at 95 °C for 3 min, followed by 26–27 cycles at 98 °C for 20 s, 58 °C for 15 s and 72 °C for 20 s. The final cycle was: 72 °C for 1 min and ending at 12 °C. The triplicate products were pooled and the concentration was measured by Qubit. The samples were diluted to an equal concentration, 1.2 ng/ul, and sent to SciLifeLab (Stockholm, Sweden) for purifications, indexing PCR, library pooling and sequencing. A 300bp paired-end sequencing run was performed using the Illumina MiSeq platform. The negative water controls did not yield PCR products and were excluded from sequencing.

#### Pre-processing and clustering of ITS reads

Reads were cut to only the desired fragment using the PCR primer sequences specified above and cutadapt (v2.4) [34]. DADA2 (v1.14) was used for filtering, trimming (modified settings: ‘maxEE = c(6,6)’), error learning, dereplication, denoising (using ‘pool = TRUE’), and merging of forward and reverse reads [35]. The ‘consensus’ method was applied for chimera removal.

Using the resulting amplicon sequence variants (ASVs), we excised the ITS2 region using ITSx (v 1.1.2) [36]. The resulting fungal ITS2 sequences were once again dereplicated using DADA2, and clustered using Swarm (v. 3.0.0) with the parameter ‘d −3’ [37]. The tool decontam (v 1.20.0) was used on resulting operational taxonomic units (OTUs) to infer likely contaminants in the negative water control sample, these were removed before further processing [38]. OTU taxonomy was assigned using constax with default parameters (v 2.0.19 [39]), UNITE was used as fungal reference database (Release 10/2022) [40].

#### Pre-processing of 16S reads

The nf-core ampliseq pipeline (v2.4.0) [41] with default settings was used to process the raw sequencing reads. Briefly, the pipeline used FastQC (v0.11.9) [42] for quality control, cutadapt (v3.4) [34] to trim the primers and DADA2 (v1.22.0) [35] to infer amplicon sequencing variants (ASVs) and taxonomically annotate them against the SILVA database (v138.1) [43].

### Statistical analyses

All further analyses were run in R (v 4.0.3) [44], unless specified otherwise. Visualizations were created with ggplot2 [45], unless mentioned. SOTUs or ASVs with fewer than three reads in at least two replicates or less than 0.005% total abundance within one condition (perfusate + litter) were removed from the dataset. Samples were rarefied to minimum sampling depth (2772 reads for ITS samples; 37679 for 16S samples) using vegan (v 2.5.6) [46] before calculating Bray-Curtis dissimilarities and performing principal coordinate analysis (PCoA), which was visualized using phyloseq (v 1.28) [42–47]. Fungal community differences between treatments were tested with permutational multivariate analysis (PERMANOVA), using the adonis function from the vegan package. Differential abundance on phylum, class, and family level was assessed using the lefser package (v 1.0.0) [43–48]. To normalise unrarefied data for visualisation purposes, the R package zinbwave (v 1.12.0) was used to normalize the data with parameter ‘K = 2’ and epsilon set to the number of samples [49]. Normalised counts were visualized using R package pheatmap [50]. To calculate observational weights for differential OTU or ASV abundances, we used zinbwave with parameters ‘K = 0, epsilon = 1e12’. The resulting observational weights were used in DESeq2 (v 1.40.2) for differential abundance analysis [51]. Based on PCoA and taxonomic composition, a specific sucrose sample (S4) from the ITS dataset was identified as an outlier (most likely from contamination) and was removed before further analyses.

### Scanning electron microscopy (SEM)

An additional set of microdialysis probe samples, prepared in the same way as described above, was prepared for scanning electron microscopy (SEM) imaging. The tubing on the probes were gently cut off with a sterile scalpel and the probes were immediately put in a fixative solution (2.5% glutaraldehyde in 0.1 M phosphate buffer) and kept at 4 °C for 7 h. Samples were then washed in a buffer solution (0.1 M phosphate). After that the samples were dehydrated in a series of ethanol gradients, critical point dried (Leica EM CPD 300) and coated with 5 nm platinum (Quorum Q150 T ES). The sample morphologies were analysed by field-emission scanning electron microscopy (FESEM, Carl Zeiss Merlin) using an in-chamber (ETD) secondary electron detector at an accelerating voltage of 5 kV and probe current of 100 pA.

**Fig. 2:**
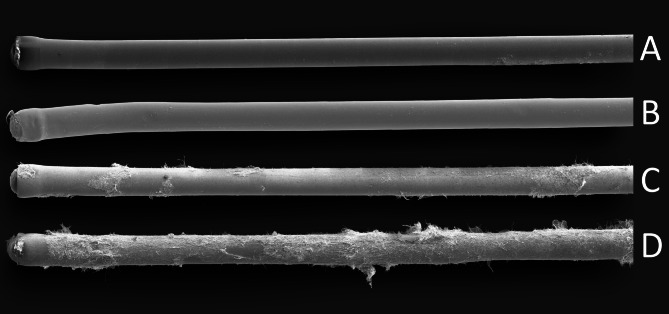
Scanning electron microscopy images of the microdialysis probes from the **A**) control treatment no litter, **B**) control treatment with litter **C**) sucrose treatment no litter and **D**) sucrose treatment with litter. Magnification 500x, the diameter of the probes is 0.5 mm. Image formatting by DC SciArt

The overview images were taken with another scanning electron microscope (SEM, Carl Zeiss EVO LS15), using a secondary electron detector with an accelerating voltage of 15 kV and probe current of 300 pA and a magnification of 500x (Fig. 2).

## Results

### Scanning electron microscopy imaging

There was a clear visual treatment effect of sucrose supply, with more visible fungal hyphae and spore-forming structures on the sucrose-releasing probes (Fig. 2). *Penicillium*-like spore-forming structures were visible, but it was not possible to identify to species level (Fig. S1, Additional File 1). In addition to the fungal hyphae, many bacterial cells were visible on the probes and on the fungal hyphae (Fig. S1, Additional File 1).

### DNA sequencing

After rarefaction and filtering there were a total of 206 fungal OTUs, of which 181 were common between the control and sucrose treatment (Table S5, Additional File 1). While fungal communities were significantly different between sucrose and control treatments (PERMANOVA *p* < 0.001), there were no treatment effects of litter for the fungal community (PERMANOVA *p* = 0.252). Thus, all further analyses of fungi were done independently of litter type, based on the perfusate treatment only (control, sucrose). Of the 181 OTUs common for both treatments (Table S5, Additional File 1), 24 had significantly differential abundance between the treatments. Fifteen OTUs were more abundant in the sucrose treatment and nine were more abundant in the control treatment (Table 1).

**Table 1:** Taxonomic annotations and summary statistics of the differentially abundant fungal OTUs between sucrose and control treatments

Phylum	Order	Genus	Species	OTU	log2 FC	padj	baseMean
Ascomycota	Hypocreales	Trichoderma	*Trichoderma viride*	OTU3	5.83	7.7E-06	322.5
Basidiomycota	Filobasidiales	Piskurozyma		OTU27	5.71	1.9E-03	40.4
Ascomycota	Eurotiales	Penicillium		OTU1	5.34	1.4E-08	3089.3
Ascomycota	Helotiales			OTU17	4.96	1.3E-05	30.3
Ascomycota	Hypocreales	Tolypocladium	*Tolypocladium album*	OTU85	4.38	2.5E-03	2.9
Ascomycota	Eurotiales	Penicillium		OTU42	4.29	3.4E-03	9.4
Mucoromycota				OTU58	4.03	8.6E-03	4.7
Ascomycota	Hypocreales	Pochonia		OTU32	3.97	3.4E-03	14.6
Ascomycota	Eurotiales	Penicillium		OTU10	3.82	3.0E-06	63.7
Ascomycota	Chaetosphaeriales			OTU37	3.17	2.5E-03	10.6
Ascomycota	Hypocreales	Trichoderma		OTU21	3.13	5.0E-03	21.4
Ascomycota	Hypocreales	Lecanicillium	*Lecanicillium flavidum*	OTU56	3.12	3.4E-03	4.7
Mucoromycota	Mucorales	Mucor	*Mucor silvaticus*	OTU6	2.08	3.8E-03	6.5
Mortierellomycota	Mortierellales	Mortierella	*Mortierella macrocystis*	OTU26	1.52	3.8E-03	15.2
Ascomycota	Eurotiales	Penicillium		OTU11	1.34	5.3E-03	119.0
Ascomycota	Helotiales	Oidiodendron		OTU13	−1.30	9.2E-03	52.5
Basidiomycota	Atheliales	Tylospora	*Tylospora asterophora*	OTU12	−1.91	2.0E-03	59.9
Ascomycota	Helotiales	Oidiodendron		OTU30	−1.94	3.8E-03	19.7
Ascomycota	Helotiales	Oidiodendron		OTU29	−2.01	2.5E-03	16.5
Ascomycota	Helotiales	Oidiodendron		OTU7	−2.02	7.4E-04	54.7
Ascomycota	Pezizales	Trichophaea		OTU4	−2.13	1.7E-03	234.5
Rozellomycota				OTU40	−2.44	5.6E-03	10.0
Ascomycota	Helotiales			OTU23	−2.67	2.5E-04	23.4
Ascomycota	Helotiales			OTU33	−2.93	2.5E-03	11.8

The upper portion of the table shows OTUs with significantly higher abundance in the sucrose treatment (log2 FC>0). OTUs with significantly higher abundance in the control treatment are shown in the lower part (log2 FC<0). Log2 FC: log2 fold change as calculated by DESeq2; padj: adjusted *p*-value; baseMean: Average of normalized count values divided by size factors, as calculated by DESeq2

The bacterial community consisted of 536 filtered ASVs, of which 181 were common for all treatments (Table S6, Additional File 1). Similar to the fungal community, the sucrose treatment (without litter) had a stimulating effect on the largest number of bacterial ASVs (13 ASVs) compared to the control treatment and only two ASVs were more abundant in the control treatment (Table 2). Contrary to the fungal communities, the bacterial communities were significantly affected both by perfusate (PERMANOVA *p* < 0.001) and by the litter treatment (PERMANOVA *p* < 0.001). The presence of litter (control with litter compared to control) increased the abundance of five ASVs of which three also were increased by sucrose and two were increased only in the control litter treatment. Comparing the control with litter and sucrose treatments including litter, two additional ASVs were increased compared to the effect of sucrose or litter alone.

**Table 2:** Taxonomic annotations and summary statistics of the differentially abundant bacterial ASVs between sucrose and control treatments

Class	Family	Genus/Species	ASVID	log2 FC	padj	BaseMean
Gammaproteobacteria	Burkholderiaceae	*B.-C.-P. caledonica*	ASV11	3.73	1.77E-03	706.2
Actinobacteria	Actinospicaceae	*Actinospica* sp.	ASV84	3.11	1.20E-03	36.6
Gammaproteobacteria	Burkholderiaceae	*Pandoraea* sp.	ASV28	3.05	1.77E-03	218.3
Gammaproteobacteria	Burkholderiaceae	*B.-C.-P. phytofirmans*	ASV82	3.02	7.25E-03	43.8
Gammaproteobacteria	Burkholderiaceae	*B.-C.-P. phenazinium*	ASV25	2.91	1.20E-03	200.4
Gammaproteobacteria	Burkholderiaceae	*B.-C.-P. phenazinium*	ASV50	2.82	7.25E-03	99.7
Gammaproteobacteria	Burkholderiaceae	*B.-C.-P. phenazinium*	ASV12	2.76	1.20E-03	653.5
Gammaproteobacteria	Burkholderiaceae	*B.-C.-P. phenazinium*	ASV9	2.76	1.77E-03	886.0
Gammaproteobacteria	Burkholderiaceae	*B.-C.-P.* sp.	ASV46	2.40	2.60E-03	91.9
Gammaproteobacteria	Burkholderiaceae	*B.-C.-P.* sp.	ASV6	2.31	1.20E-03	1206.4
Gammaproteobacteria	Burkholderiaceae	*Pandoraea* sp.	ASV1	2.03	7.05E-03	11643.9
Acidobacteriae	Acidobacteriaceae (SG 1)	*Granulicella* sp.	ASV87	1.88	5.74E-03	39.3
Acidobacteriae	Acidobacteriaceae (SG 1)	*Granulicella* sp.	ASV78	1.44	7.05E-03	41.7
Alphaproteobacteria	Xanthobacteraceae	*Bradyrhizobium elkanii*	ASV26	−1.29	6.79E-03	150.4
Gammaproteobacteria	Oxalobacteraceae		ASV3	−1.94	6.79E-03	2352.0

The upper portion of the table shows ASVs with significantly higher abundance in the sucrose treatment (log2 FC>0), while the lower part of the table shows ASVs significantly higher in abundance in the control treatment (log2 FC<0). The genus complex *Burkholderia-Caballeronia-Paraburkholderia* has been abbreviated to B.-C.-P. Log2 FC: log2 fold change as calculated by DESeq2; padj: adjusted *p*-value; baseMean: Average of normalized count values divided by size factors, as calculated by DESeq2

Fungal richness (Wilcoxon rank sum test, *p* < 0.001; Fig. S2 C, Additional File 1) and Shannon diversity index (Wilcoxon rank sum test, *p* < 0.001; Fig. S2 A, Additional File 1) were significantly higher and showed smaller variation in the control treatment than in sucrose treatment, while presence of litter had no effect on fungal alpha diversity. The richness of bacteria was not significantly affected by the sucrose treatment, but the combination of sucrose and litter resulted in significantly lower richness than the other treatment combinations (Fig. S2D, Additional File 1). Shannon diversity indices showed a similar pattern, with only the combination of litter and sucrose resulting in a significantly lowered Shannon diversity index compared to control without litter (Fig. S2B, Additional File 1).

**Fig. 3:**
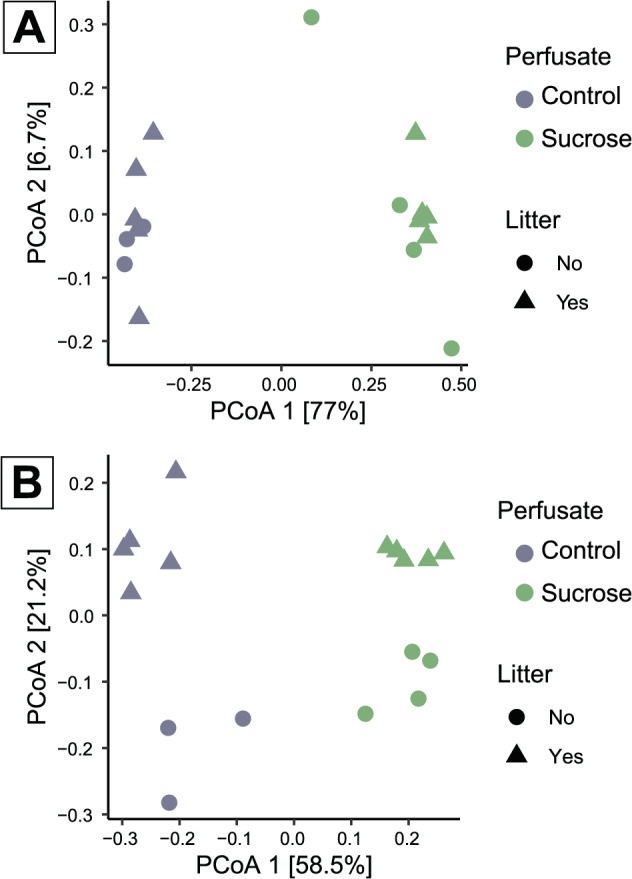
PCoA plot of **A**) OTUs on fungal composition on the microdialysis membranes and **B**) ASVs on bacterial composition. For the treatments control and sucrose with and without litter application. *N* = 3–5

The PCoA plot showed a clear difference in fungal community structure between the control and sucrose treatment. Principal coordinate 1, which explained 77% of the variation, clearly separated samples from the two treatments (Fig. 3A, PERMANOVA *p* < 0.001). The PCoA plot for the bacterial community structure, similarly to the fungal community, showed clear differences between the control and sucrose treatment (PERMANOVA *p* < 0.001). Principal coordinate 1 explained 59% of the variation and clearly separated samples from the two treatments (Fig. 3B). Contrary to the fungal community, the bacterial community structure showed a clear and significant (PERMANOVA *p* < 0.001) separation between samples with and without litter, with principal coordinate 2 explaining 21% of the variation.

### Differences at phylum, class and family level

#### Fungi

At the fungal phylum level, Ascomycota dominated all treatments (70–90% of reads) and had consistently higher relative abundances in the sucrose treatment compared to the control treatment, (Fig. S3 A, Additional File 1). Basidiomycota displayed an opposite trend, with significantly higher relative abundance in the control treatment than the sucrose treatment (Fig. S3 A, Additional File 1).

**Fig. 4:**
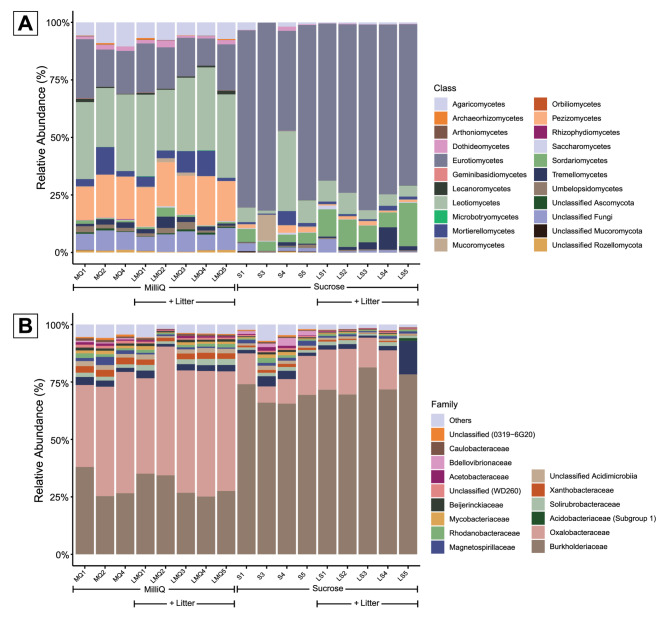
Stacked bar graphs showing **A**) differences in fungal composition at class level and **B**) bacterial community compositions on family level for the 15 most abundant families. For the treatments control and sucrose with and without litter application. *N* = 3–5

Consistent to the differences at the phylum level, two classes of Ascomycota; Eurotiomycetes and Sordariomycetes, had a higher abundance in the sucrose treatment compared to the control treatment (Fig. 4A). Eurotiomycetes composed the largest difference (23% in control treatment and 76% average relative abundance in the sucrose treatment) (Clustered heat map of classes; Fig. S4 A, Additional File 1). Also, in agreement with differences at phylum level the Basidiomycota class Agaricomycetes and the Mortierellomycota class, Mortierellomycetes, had a higher abundance in the control treatment compared to the sucrose treatment. In addition, opposite to the trend of Ascomycota being more abundant in the sucrose treatment, two Ascomycota classes, Pezizomycetes and Leotiomycetes had more reads in the control treatment compared to the sucrose treatment (Fig. S4 A, Additional File 1).

Among the OTUs stimulated by the sucrose treatment, there were several members of the genera *Penicillium*, *Trichoderma*, *Mucor*, and *Mortierella*. Species that were significantly reduced in abundance included members of the genera *Oidiodendron*, *Trichophaea*, and *Tylospora* (Table 1).

#### Bacteria

At the bacterial phylum level, Proteobacteria dominated all treatments (80–90% of reads) and had significantly higher abundances in the litter treatment, while Bdellovibrionota had significantly lower abundance in the litter treatment, mainly due to treatment unique ASVs in the control treatment (Fig. S3B, Additional File 1). At family level, we observed a strong increase in relative abundance for Burkholderiaceae at the cost of mostly Oxalobacteraceae when sucrose was used as perfusate (Fig. 4B).

Similar to the differences at family level, the majority of ASVs found to be significantly more abundant in the sucrose treatment were assigned to the genus complex *Burkholderia-Caballeronia-Paraburkholderia*, and *Pandoraea* (Burkholderiaceae) and in addition the genus *Actinospica* (Actinospicaceae) (Table 2; Fig. S4B, Additional File 1). Only two ASVs were significantly more abundant in absence of sucrose (control treatment) assigned to the genus *Bradyrhizobium* and the family Oxalobacteraceae. The presence of litter (control with litter) also significantly increased abundances of two *Burkholderia-Caballeronia-Paraburkholderia* ASVs (same ASVs as affected by the sucrose treatment) and three ASVs assigned to Burkholderiaceae, *Conexibacter*, and *Pandoraea* (also in the sucrose treatment), while only two ASVs assigned to *Undibacterium* were less abundant in the litter treatment (Table S3, Additional File 1). The addition of litter did not have a large effect on differentially abundant ASVs between control and sucrose treatments. Several ASVs were more abundant in the control litter treatment but their abundances were very low in comparison to the other ASVs (Table S4, Additional File 1).

## Discussion

By the release of carbon in the form sucrose over four days, we were able to significantly stimulate microbial growth on the microdialysis probe surface from the organic layer of boreal forest soil. Fungal hyphae and bacterial cells were clearly seen on the SEM images (Fig. 2C; Fig. S1, Additional File 1). Litter addition did not result in any visible differences in the SEM images (Fig. 2B) and also did not affect fungal species composition at the probe surface. However, the addition of litter decreased bacterial alpha diversity (Fig. S2B/D, Additional File 1) and changed bacterial community composition (Fig. 3B) compared to the no litter treatment, but to a lesser extent than the sucrose treatment. Previous studies on decomposition processes have shown that lower substrate carbon/nitrogen ratios increase bacterial growth relative to fungal growth [52, 53] which may have contributed to the litter addition treatment only affecting bacterial alpha diversity and community composition during the relatively short duration of the experiment.

Most of the fungal species significantly stimulated by the sucrose were classified as fast growing or opportunistic, e.g. yeasts (Tremellomycetes, Table 1) [54] and moulds (*Penicillium* spp., *Mucor sylvaticus*), which are known to respond quickly (within days) to simple sugars in the form of root exudates in the rhizosphere [55]. An unidentified *Penicillium* had the largest relative abundance and largest difference between the sucrose treatment compared to the control treatment (Table 1; Fig. S5, Additional File 1). All the species that were affected the most by the sucrose treatment have been found in rhizospheric soil and in the humic layer in forest soils, such as the substrate used in the present study and on tree roots from nearby sites (*Penicillium* spp., *Trichoderma*,* Mucor sylvaticus*, Burkholderiaceae, Actinospicaceae) [32, 56–58]. While lack of resolution within the ITS2 region did not allow us to identify all OTUs to species level, the most abundant OTU matched well to *Penicillium spinulosum*. This *Penicillium* species has previously been shown to be the third most common species in both managed and unmanaged *Pinus sylvestris* forest soil and has been found on both Scots pine and Norway spruce roots in nurseries [59, 60]. Based on the compound released and the short time scale, these results were as expected [55]. Both *Penicillium spinulosum* and various species of *Trichoderma* have been identified as showing invertase activity, an enzyme required to hydrolyse sucrose [61, 62], which would indicate that they can utilise sucrose for growth, unlike ectomycorrhizal fungal species [63]. Many bacterial species in Proteobacteria, the dominant phylum affected by the sucrose release, are referred to as quickly responsive to labile carbon [57] and members of some families (Burkholderiaceae and Actinospicaceae) can survive on sucrose as a sole carbon source, indicating invertase activity [64]. Some of the species with invertase activity also have extracellular invertases, which could explain the production of glucose and fructose measured after release of sucrose in a previous study using the same experimental setting [20].

Several taxa had significantly higher abundance in the control treatment compared to the sucrose treatment (*Oidiodendron* spp, *Trichophaea* sp., *Tylospora asterophora*, Table 1, Oxobacteraceae, *Bradyrhizobium elkanii*, Table 2). The control treatment also had more treatment specific OTUs and ASVs than the sucrose treatment. Among these were several ectomycorrhizal species (3 *Cortinarius* species, *Piloderma olivaceum*) (Table S5, Additional File 1). Mycorrhizal forming species were also found on the sucrose treatment probes (*Cortinarius*,* Piloderma*,* Tylospora*,* Lactarius*,* Cenococcum*) (Table S5, Additional File 1), which are all species commonly found in these soils [56] but none of these species were significantly stimulated by the treatment. This was not unexpected since most ectomycorrhizal fungi do not have the ability to directly take up sucrose as a carbon source [63]. Still, as mentioned earlier, production of glucose, which is one of the preferred carbon sources for ectomycorrhizal fungi, has been detected during the release of sucrose with the same experimental set up as in the present study [20]. The low abundance of mycorrhizal species undoubtedly also results from the slow growth rate of mycorrhizal fungi and the short duration of the experiment (four days), as well as the disturbance and homogenisation of the soil prior to the start of the experiment and the storage of soil in absence of living roots (ectomycorrhizal carbon supply). Considering the very low visible growth on the control probes in the SEM images, it is likely that many of the species detected by sequencing were sequenced from relic DNA of fungi and bacteria inhabiting the soil before the soil was taken from the forest, as relic DNA from dead cells can constitute a significant portion of environmental DNA [65]. Moreover, the decreased relative abundance of Basidiomycota in sucrose samples is likely a consequence of increased growth of opportunistic Ascomycetes in sucrose samples, due to the compositional nature of amplicon sequencing data [66]. In future studies, RNA-seq based approaches can be used to focus on actively growing microbes.

## Conclusions

In the present study, we show that microdialysis can be used to study fungal and bacterial growth response to the release of simple carbon compounds, demonstrating the potential of this technique in unravelling microbial interactions in connection to carbon exudation. In a broader view, the technique can be used as a study system for the response of microbes to separate compounds of root exudates on a microscopic scale. The uniqueness of using microdialysis to study microbial interactions is the possibility to not only release a compound of interest, but to also follow the resulting effect on microbial community composition and activity. It enables sampling of the chemical response from the microbes within a short (minutes-hours-days) time scale, as we have previously demonstrated in Buckley et al. [20]. In practise, this means that simultaneous release and sampling of chemical responses is possible, in soil and other microbial environments.

## Supplementary Information

Additional File 1. This document contains Figures S1– S5 and Tables S1– S8 including legend texts.

## Data Availability

The DNA amplicon sequencing data supporting the conclusions of this article are available in the European Nucleotide Archive (ENA) with the accession PRJEB61550, https://www.ebi.ac.uk/ena/browser/view/PRJEB61550. The R code used for processing and analysis of the data can be accessed on GitHub [67].

## Abbreviations

ASV: Amplicon sequence variant
ITS: Internal transcribed spacer
MQ: Milli-Q water (de-ionised)
OTU: Operational taxnomic unit
PCoA: Principal coordinate analysis
