# Supplementary material for: Reverse microdialysis of sucrose stimulates soil fungal and bacterial growth at the microscale
Source: BMC Microbiol. 2025 Jul 14;25:436. doi: 10.1186/s12866-025-04082-5 (PMC12257659; doi:10.1186/s12866-025-04082-5)
Supplement: Supplementary file 1 — Additional File 1. This document contains Figures S1– S5 and Tables S1– S8 including legend texts. [file 12866_2025_4082_MOESM1_ESM.docx]

**SUPPLEMENTARY MATERIAL**

**
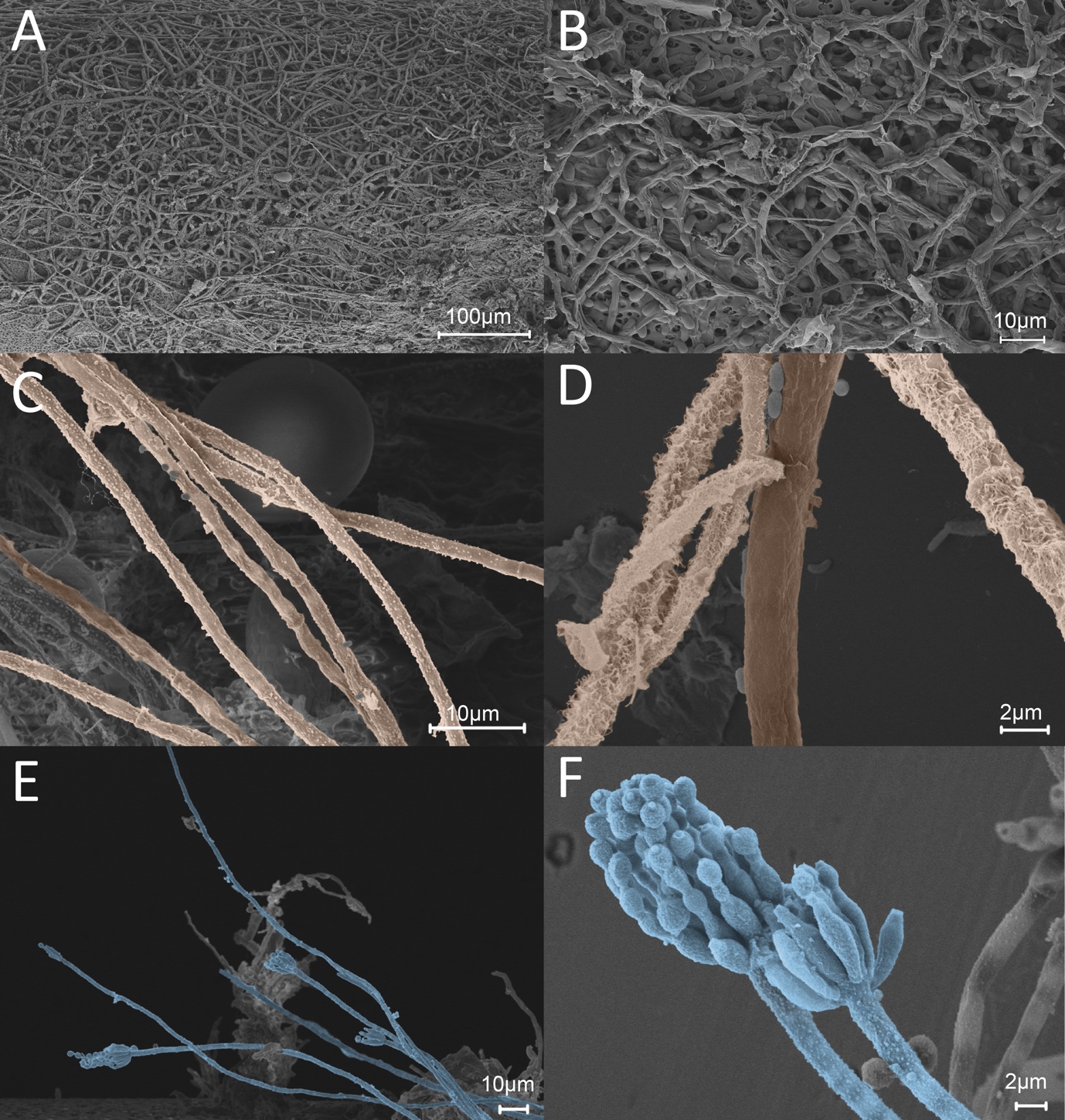
**

**Fig. S1.** Close up SEM images of microbial structures on the sucrose treatment probes. Note the different scales on the different pictures. A, B) Fungal hyphal mat on the probe. C, D) Fungal hyphae with different surface structures due to anatomy or artefacts by sample fixation C) with what could potentially be a testate amoeba in the background and C, D) what could be rod shaped bacteria on the hyphae. E, F) Penicillium-like spore forming structures. Image formatting by DC SciArt.

**
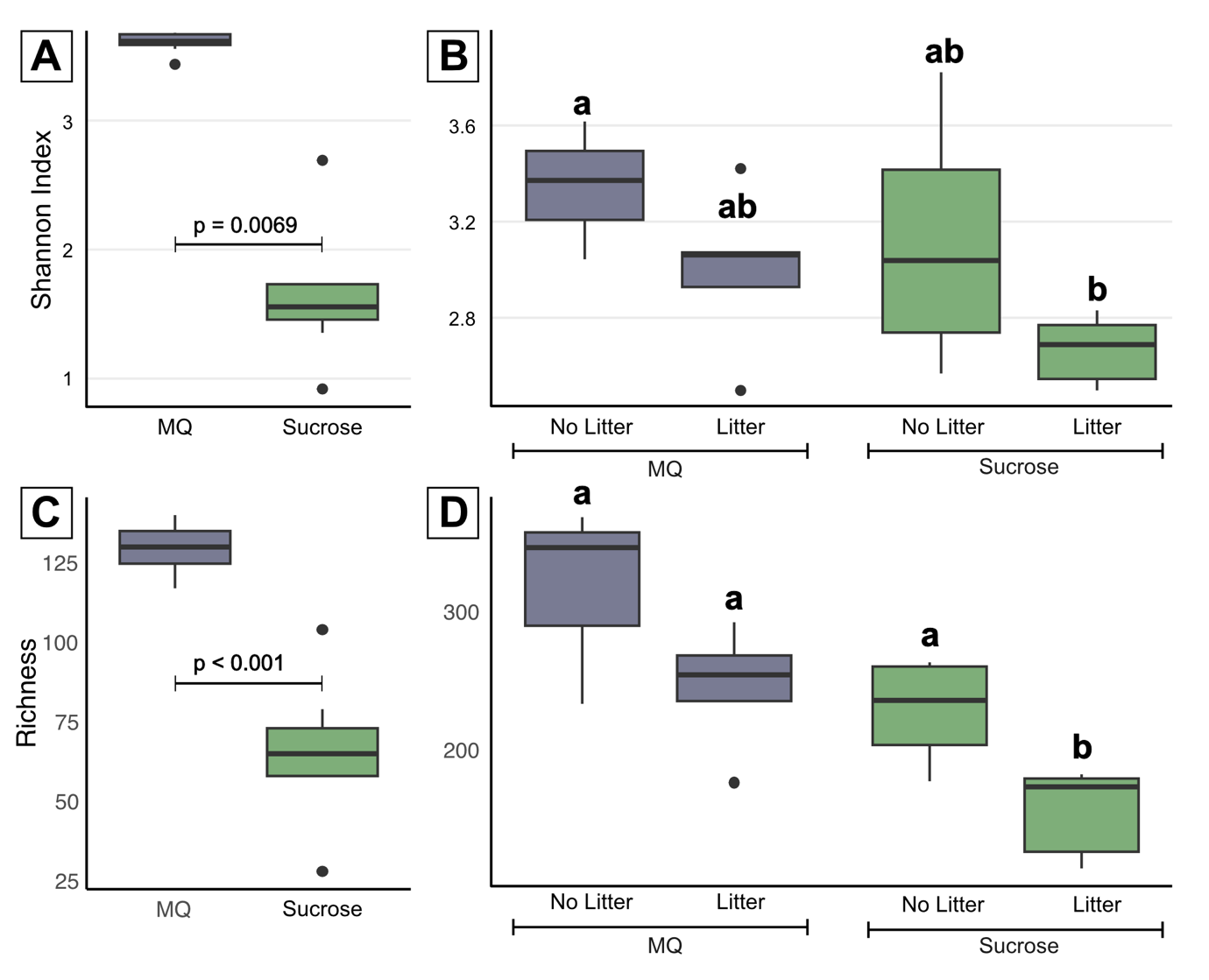
**

**Fig. S2.** Alpha diversity of fungal and bacterial communities, with (A) and (B) showing Shannon diversity index values for fungal OTUs and bacterial ASVs, respectively. (C) and (D) show richness (excluding singletons) of fungal OTUs and bacterial ASVs. Significance was calculated in R using Wilcoxon rank sum test (A,C), and the Kruskal-Wallis test as implemented in the agricolae package (B,D).


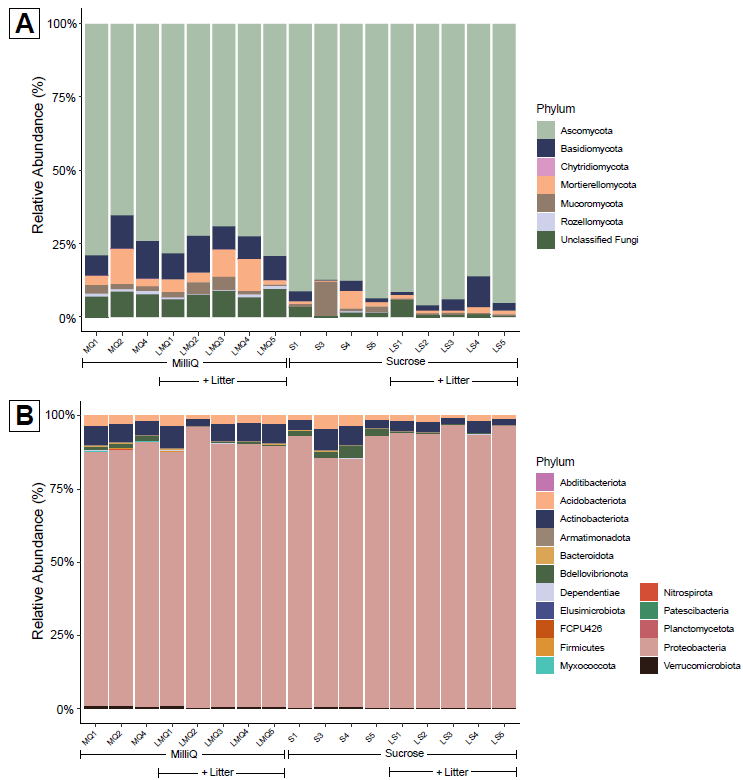


**Fig. S3.** Phylum level distribution for the different treatments for A) fungal OTUs and B) bacterial ASVs.


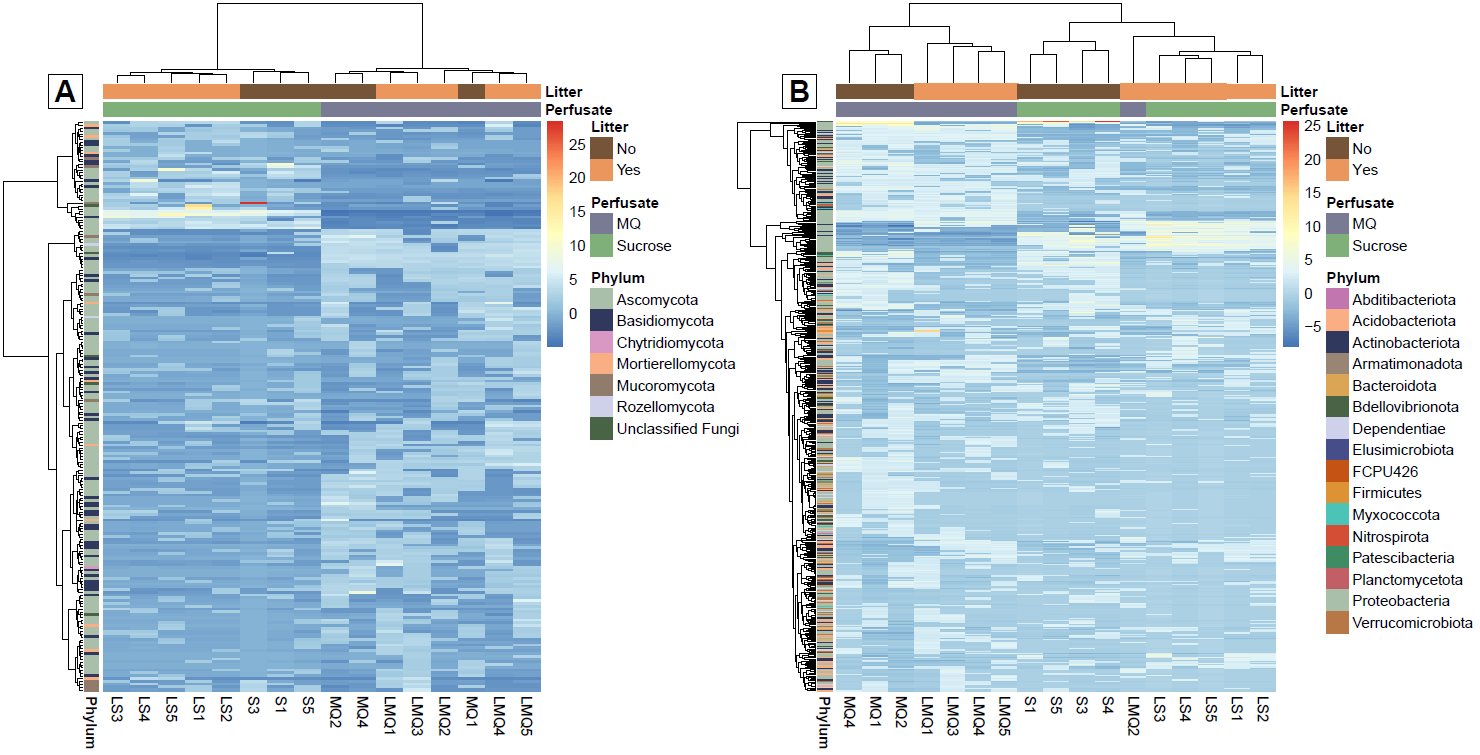


**Fig. S4.** Clustered heat map of normalized values to give an overview of treatment effects on A) fungal OTUs and B) bacterial ASVs. Coloured bar left of the plot is highlighting their respective phyla.

**
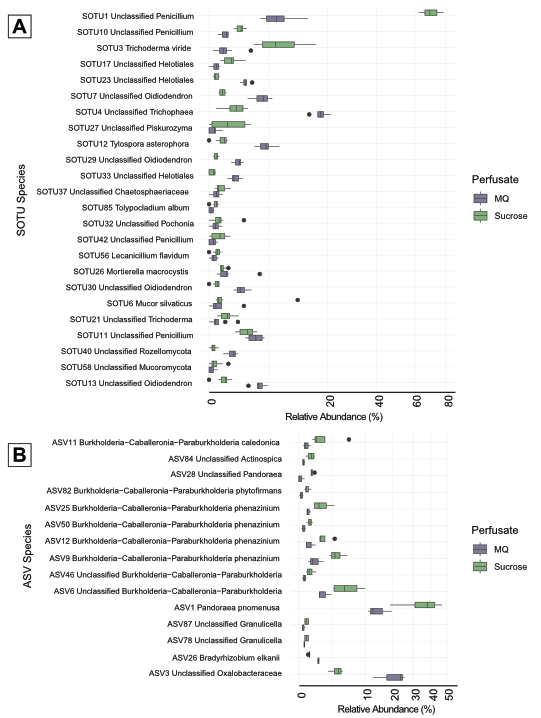
**

**Fig. S5.** Boxplots showing relative abundances of significantly differentially abundant fungal OTUs (**A**) and bacterial ASVs (**B**) between sucrose and control (“MQ”) samples.

**Table S1.** Sequences of primers used in the amplicon sequencing of ITS2. gITS7 and ITS4 were used in PCR 1, the primers displayed consist of the overhang and the locus specific sequence, separated by Ns. Index primers were used in PCR 2, and consist of P5/P7 elements for Illumina sequencing, an 8bp barcode (XXXXXXXX), and a complement to the overhang from PCR 1.

| **Oligo Name** | **Sequence** (written 5' - 3') |
| --- | --- |
|  |  |
| gITS7 | ACACTCTTTCCCTACACGACGCTCTTCCGATCTGTGARTCATCGARTCTTTG |
| gITS7N | ACACTCTTTCCCTACACGACGCTCTTCCGATCTNGTGARTCATCGARTCTTTG |
| gITS7NN | ACACTCTTTCCCTACACGACGCTCTTCCGATCTNNGTGARTCATCGARTCTTTG |
| gITS7NNN | ACACTCTTTCCCTACACGACGCTCTTCCGATCTNNNGTGARTCATCGARTCTTTG |
| ITS4 | GTGACTGGAGTTCAGACGTGTGCTCTTCCGATCTTCCTCCGCTTATTGATATGC |
| ITS4N | GTGACTGGAGTTCAGACGTGTGCTCTTCCGATCTNTCCTCCGCTTATTGATATGC |
| ITS4NN | GTGACTGGAGTTCAGACGTGTGCTCTTCCGATCTNNTCCTCCGCTTATTGATATGC |
| ITS4NNN | GTGACTGGAGTTCAGACGTGTGCTCTTCCGATCTNNNTCCTCCGCTTATTGATATGC |
| Index1 | CAAGCAGAAGACGGCATACGAGATXXXXXXXXGTCTCGTGGGCTCGG |
| Index2 | AATGATACGGCGACCACCGAGATCTACACXXXXXXXXTCGTCGGCAGCGTC |

**Table S2.** Sequences of primers 799F and 1193R used in the amplicon sequencing of the V5-V7 region of the 16S rRNA gene. The primers displayed consist of the overhangs compatible with index primers and the locus specific sequence, separated by Ns.

| **Oligo Name** | **Sequence** (written 5' - 3') |
| --- | --- |
|  |  |
| 799F | ACACTCTTTCCCTACACGACGCTCTTCCGATCTAACMGGATTAGATACCCKG |
| 799FN | ACACTCTTTCCCTACACGACGCTCTTCCGATCTNAACMGGATTAGATACCCKG |
| 799FNN | ACACTCTTTCCCTACACGACGCTCTTCCGATCTNNAACMGGATTAGATACCCKG |
| 799FNNN | ACACTCTTTCCCTACACGACGCTCTTCCGATCTNNNAACMGGATTAGATACCCKG |
| 1193R | GTGACTGGAGTTCAGACGTGTGCTCTTCCGATCTACGTCATCCCCACCTTCC |
| 1193RN | GTGACTGGAGTTCAGACGTGTGCTCTTCCGATCTNACGTCATCCCCACCTTCC |
| 1193RNN | GTGACTGGAGTTCAGACGTGTGCTCTTCCGATCTNNACGTCATCCCCACCTTCC |
| 1193RNNN | GTGACTGGAGTTCAGACGTGTGCTCTTCCGATCTNNNACGTCATCCCCACCTTCC |

**Table S3.** Taxonomic annotations of the differentially abundant ASVs between MQ and MQ plus litter treatments. In dark blue, 5 ASVs with significantly higher number of reads in the MQ plus litter treatment and 2 ASVs significantly higher in number of reads in the MQ treatment in lighter blue. The genus complex Burkholderia-Caballeronia-Paraburkholderia has been abbreviated to B.-C.-P.

| **Phylum** | **Class** | **Order** | **Family** | **Genus** | **Species** | **ASV_ID** | **log2FC** | **padj** | **baseMean** |
| --- | --- | --- | --- | --- | --- | --- | --- | --- | --- |
| Proteobacteria | Gammaproteobacteria | Burkholderiales | Burkholderiaceae | B.-C.-P. | *B.-C.-P. phenazinium* | ASV_11 | 3.62 | 3.19E-03 | 706.2 |
| Proteobacteria | Gammaproteobacteria | Burkholderiales | Burkholderiaceae | B.-C.-P. | *B.-C.-P. phenazinium* | ASV_12 | 3.33 | 3.46E-04 | 653.5 |
| Proteobacteria | Gammaproteobacteria | Burkholderiales | Burkholderiaceae | Pandoraea |  | ASV_28 | 2.60 | 7.70E-03 | 218.3 |
| Proteobacteria | Gammaproteobacteria | Burkholderiales | Burkholderiaceae |  |  | ASV_10 | 2.52 | 6.42E-04 | 741.0 |
| Actinobacteriota | Thermoleophilia | Solirubrobacterales | Solirubrobacteraceae | Conexibacter |  | ASV_47 | 2.37 | 4.04E-03 | 92.6 |
| Proteobacteria | Gammaproteobacteria | Burkholderiales | Oxalobacteraceae | Undibacterium |  | ASV_27 | -2.77 | 4.44E-04 | 139.5 |
| Proteobacteria | Gammaproteobacteria | Burkholderiales | Oxalobacteraceae | Undibacterium |  | ASV_31 | -4.24 | 3.46E-04 | 104.4 |

**Table S4.** Taxonomic annotations of the differentially abundant ASVs between sucrose plus litter and MQ plus litter treatments. In dark green, 8 ASVs with significantly higher number of reads in the sucrose plus litter treatment and 13 ASVs significantly higher in number of reads in the MQ plus litter treatment in dark blue. The genus complex Burkholderia-Caballeronia-Paraburkholderia has been abbreviated to B.-C.-P.

| **Phylum** | **Class** | **Order** | **Family** | **Genus** | **Species** | **ASV_ID** | **log2FoldChange** | **padj** | **baseMean** |
| --- | --- | --- | --- | --- | --- | --- | --- | --- | --- |
| Actinobacteriota | Actinobacteria | Catenulisporales | Actinospicaceae | Actinospica |  | ASV_176 | 11.31 | 7.74E-03 | 8.4 |
| Proteobacteria | Gammaproteobacteria | Burkholderiales | Burkholderiaceae | Pandoraea |  | ASV_7 | 2.74 | 2.47E-03 | 1336.1 |
| Proteobacteria | Gammaproteobacteria | Burkholderiales | Burkholderiaceae | Pandoraea |  | ASV_1 | 2.60 | 2.13E-03 | 11643.9 |
| Actinobacteriota | Actinobacteria | Catenulisporales | Actinospicaceae | Actinospica |  | ASV_84 | 2.51 | 4.68E-03 | 36.6 |
| Proteobacteria | Gammaproteobacteria | Burkholderiales | Burkholderiaceae | Pandoraea |  | ASV_28 | 2.41 | 5.42E-04 | 218.3 |
| Proteobacteria | Gammaproteobacteria | Burkholderiales | Burkholderiaceae | B.-C.-P. | *B.-C.-P. phenazinium* | ASV_9 | 2.29 | 3.79E-03 | 886.0 |
| Proteobacteria | Gammaproteobacteria | Burkholderiales | Burkholderiaceae | B.-C.-P. |  | ASV_6 | 2.11 | 2.13E-03 | 1206.4 |
| Proteobacteria | Gammaproteobacteria | Burkholderiales | Burkholderiaceae | B.-C.-P. |  | ASV_5 | 1.74 | 6.51E-03 | 1160.6 |
| Proteobacteria | Alphaproteobacteria | Rhizobiales | Xanthobacteraceae | Bradyrhizobium | *Bradyrhizobium elkanii* | ASV_26 | -1.48 | 5.18E-03 | 150.4 |
| Proteobacteria | Gammaproteobacteria | Burkholderiales | Oxalobacteraceae |  |  | ASV_8 | -1.68 | 5.18E-03 | 832.2 |
| Proteobacteria | Gammaproteobacteria | Burkholderiales | Oxalobacteraceae |  |  | ASV_3 | -1.84 | 5.18E-03 | 2352.0 |
| Proteobacteria | Alphaproteobacteria | Rhizobiales | Beijerinckiaceae |  |  | ASV_74 | -1.86 | 2.29E-03 | 37.0 |
| Actinobacteriota | Actinobacteria | Corynebacteriales | Mycobacteriaceae | Mycobacterium |  | ASV_138 | -10.66 | 3.79E-03 | 11.7 |
| Proteobacteria | Alphaproteobacteria | Rhizobiales | Beijerinckiaceae | Roseiarcus | *Roseiarcus fermentans* | ASV_132 | -11.07 | 4.21E-03 | 11.6 |
| Myxococcota | Myxococcia | Myxococcales | Anaeromyxobacteraceae | Anaeromyxobacter |  | ASV_162 | -11.10 | 2.88E-03 | 9.1 |
| Proteobacteria | Alphaproteobacteria | Rhizobiales | Xanthobacteraceae |  |  | ASV_166 | -12.18 | 2.29E-03 | 7.5 |
| Proteobacteria | Alphaproteobacteria | Micropepsales | Micropepsaceae |  |  | ASV_248 | -12.81 | 3.43E-03 | 3.7 |
| Proteobacteria | Gammaproteobacteria | Xanthomonadales | Xanthomonadaceae | Lysobacter |  | ASV_258 | -12.85 | 2.88E-03 | 3.6 |
| Actinobacteriota | Actinobacteria | Frankiales | Acidothermaceae | Acidothermus |  | ASV_192 | -13.17 | 2.88E-03 | 6.1 |
| Proteobacteria | Alphaproteobacteria | Acetobacterales | Acetobacteraceae | Acidocella |  | ASV_245 | -13.42 | 6.18E-03 | 4.0 |
| Proteobacteria | Alphaproteobacteria | Acetobacterales | Acetobacteraceae |  |  | ASV_272 | -14.91 | 7.32E-03 | 3.4 |

**Table S5.** OTUs (181) occurring in both treatments (MQ, sucrose).

| **Phylum** | **Class** | **Order** | **Family** | **Genus** | **Species** | **OTU** |
| --- | --- | --- | --- | --- | --- | --- |
| Ascomycota | Archaeorhizomycetes | Archaeorhizomycetales | Archaeorhizomycetaceae | Archaeorhizomyces | *Archaeorhizomyces finlayi* | OTU116 |
| Ascomycota | Archaeorhizomycetes | Archaeorhizomycetales | Archaeorhizomycetaceae | Archaeorhizomyces |  | OTU126 |
| Ascomycota | Arthoniomycetes | Lichenostigmatales |  |  |  | OTU119 |
| Ascomycota | Dothideomycetes | Capnodiales | Cladosporiaceae | Cladosporium |  | OTU68 |
| Ascomycota | Dothideomycetes | Capnodiales |  |  |  | OTU140 |
| Ascomycota | Dothideomycetes | Dothideales | Dothioraceae | Hormonema | *Hormonema macrosporum* | OTU75 |
| Ascomycota | Dothideomycetes | Dothideales | Dothioraceae | Perusta | *Perusta inaequalis* | OTU177 |
| Ascomycota | Dothideomycetes | Dothideales | Saccotheciaceae | Aureobasidium | *Aureobasidium pullulans* | OTU229 |
| Ascomycota | Dothideomycetes | Mycosphaerellales | Teratosphaeriaceae | Lapidomyces |  | OTU112 |
| Ascomycota | Dothideomycetes | Mycosphaerellales | Teratosphaeriaceae | Lapidomyces |  | OTU123 |
| Ascomycota | Dothideomycetes | Mytilinidales | Gloniaceae | Cenococcum |  | OTU146 |
| Ascomycota | Dothideomycetes | Mytilinidiales | Mytilinidiaceae | Mytilinidion |  | OTU169 |
| Ascomycota | Dothideomycetes | Phaeothecales | Phaeothecaceae | Phaeotheca | *Phaeotheca fissurella* | OTU100 |
| Ascomycota | Dothideomycetes | Phaeothecales | Phaeothecaceae | Phaeotheca | *Phaeotheca fissurella* | OTU114 |
| Ascomycota | Dothideomycetes | Phaeothecales | Phaeothecaceae | Phaeotheca | *Phaeotheca fissurella* | OTU188 |
| Ascomycota | Dothideomycetes | Phaeothecales | Phaeothecaceae | Phaeotheca | *Unclassified.Phaeotheca* | OTU180 |
| Ascomycota | Dothideomycetes | Pleosporales | Didymellaceae |  |  | OTU115 |
| Ascomycota | Dothideomycetes | Pleosporales | Didymosphaeriaceae | Paraphaeosphaeria |  | OTU213 |
| Ascomycota | Dothideomycetes | Venturiales | Venturiaceae | Venturia |  | OTU49 |
| Ascomycota | Dothideomycetes | Venturiales | Venturiaceae | Venturia |  | OTU176 |
| Ascomycota | Dothideomycetes | Venturiales | Venturiaceae | Venturia |  | OTU201 |
| Ascomycota | Eurotiomycetes | Chaetothyriales | Herpotrichiellaceae | Capronia |  | OTU179 |
| Ascomycota | Eurotiomycetes | Chaetothyriales | Herpotrichiellaceae | Cladophialophora |  | OTU51 |
| Ascomycota | Eurotiomycetes | Chaetothyriales | Herpotrichiellaceae | Cladophialophora |  | OTU65 |
| Ascomycota | Eurotiomycetes | Chaetothyriales | Herpotrichiellaceae | Cladophialophora |  | OTU93 |
| Ascomycota | Eurotiomycetes | Chaetothyriales | Herpotrichiellaceae | Cladophialophora |  | OTU97 |
| Ascomycota | Eurotiomycetes | Chaetothyriales | Herpotrichiellaceae | Cladophialophora |  | OTU109 |
| Ascomycota | Eurotiomycetes | Chaetothyriales | Herpotrichiellaceae | Cladophialophora |  | OTU127 |
| Ascomycota | Eurotiomycetes | Chaetothyriales | Herpotrichiellaceae | Cladophialophora |  | OTU157 |
| Ascomycota | Eurotiomycetes | Chaetothyriales | Herpotrichiellaceae | Cladophialophora |  | OTU193 |
| Ascomycota | Eurotiomycetes | Chaetothyriales | Herpotrichiellaceae | Exophiala | *Exophiala moniliae* | OTU134 |
| Ascomycota | Eurotiomycetes | Chaetothyriales | Herpotrichiellaceae | Sorocybe |  | OTU147 |
| Ascomycota | Eurotiomycetes | Chaetothyriales | Herpotrichiellaceae |  |  | OTU36 |
| Ascomycota | Eurotiomycetes | Chaetothyriales | Herpotrichiellaceae |  |  | OTU76 |
| Ascomycota | Eurotiomycetes | Chaetothyriales | Herpotrichiellaceae |  |  | OTU110 |
| Ascomycota | Eurotiomycetes | Chaetothyriales | Herpotrichiellaceae |  |  | OTU172 |
| Ascomycota | Eurotiomycetes | Chaetothyriales |  |  |  | OTU84 |
| Ascomycota | Eurotiomycetes | Chaetothyriales |  |  |  | OTU132 |
| Ascomycota | Eurotiomycetes | Chaetothyriales |  |  |  | OTU168 |
| Ascomycota | Eurotiomycetes | Eurotiales | Aspergillaceae | Penicillium | *Penicillium atrosanguineum* | OTU79 |
| Ascomycota | Eurotiomycetes | Eurotiales | Aspergillaceae | Penicillium | *Penicillium brevicompactum* | OTU209 |
| Ascomycota | Eurotiomycetes | Eurotiales | Aspergillaceae | Penicillium | *Penicillium simplicissimum* | OTU66 |
| Ascomycota | Eurotiomycetes | Eurotiales | Aspergillaceae | Penicillium |  | OTU1 |
| Ascomycota | Eurotiomycetes | Eurotiales | Aspergillaceae | Penicillium |  | OTU10 |
| Ascomycota | Eurotiomycetes | Eurotiales | Aspergillaceae | Penicillium |  | OTU11 |
| Ascomycota | Eurotiomycetes | Eurotiales | Aspergillaceae | Penicillium |  | OTU14 |
| Ascomycota | Eurotiomycetes | Eurotiales | Aspergillaceae | Penicillium |  | OTU42 |
| Ascomycota | Eurotiomycetes | Eurotiales | Aspergillaceae | Penicillium |  | OTU69 |
| Ascomycota | Eurotiomycetes | Eurotiales | Aspergillaceae | Penicillium |  | OTU87 |
| Ascomycota | Eurotiomycetes | Eurotiales | Aspergillaceae | Penicillium |  | OTU96 |
| Ascomycota | Eurotiomycetes | Eurotiales | Aspergillaceae | Penicillium |  | OTU102 |
| Ascomycota | Eurotiomycetes | Eurotiales | Aspergillaceae | Penicillium |  | OTU103 |
| Ascomycota | Eurotiomycetes | Eurotiales | Aspergillaceae | Talaromyces |  | OTU131 |
| Ascomycota | Eurotiomycetes | Eurotiales | Trichocomaceae | Sagenomella | *Sagenomella diversispora* | OTU98 |
| Ascomycota | Eurotiomycetes | Eurotiales | Trichocomaceae | Sagenomella | *Sagenomella verticillata* | OTU99 |
| Ascomycota | Eurotiomycetes | Sclerococcales | Sclerococcaceae | Sclerococcum |  | OTU46 |
| Ascomycota | Lecanoromycetes | Acarosporales | Acarosporaceae | Acarospora |  | OTU44 |
| Ascomycota | Lecanoromycetes | Pertusariales | Megasporaceae | Aspicilia |  | OTU181 |
| Ascomycota | Lecanoromycetes |  |  |  |  | OTU107 |
| Ascomycota | Leotiomycetes | Helotiales | Dermateaceae | Cryptosporiopsis |  | OTU67 |
| Ascomycota | Leotiomycetes | Helotiales | Helotiaceae | Ascocoryne | *Ascocoryne albida* | OTU194 |
| Ascomycota | Leotiomycetes | Helotiales | Helotiaceae | Infundichalara | *Infundichalara minuta* | OTU61 |
| Ascomycota | Leotiomycetes | Helotiales | Helotiaceae | Infundichalara | *Infundichalara minuta* | OTU122 |
| Ascomycota | Leotiomycetes | Helotiales | Helotiaceae |  |  | OTU175 |
| Ascomycota | Leotiomycetes | Helotiales | Helotiaceae |  |  | OTU186 |
| Ascomycota | Leotiomycetes | Helotiales | Hyaloscyphaceae | Arachnopeziza |  | OTU150 |
| Ascomycota | Leotiomycetes | Helotiales | Hyaloscyphaceae | Hyaloscypha |  | OTU8 |
| Ascomycota | Leotiomycetes | Helotiales | Hyaloscyphaceae | Hyaloscypha |  | OTU48 |
| Ascomycota | Leotiomycetes | Helotiales | Leotiaceae | Pezoloma |  | OTU86 |
| Ascomycota | Leotiomycetes | Helotiales | Myxotrichaceae | Oidiodendron | *Oidiodendron eucalypti* | OTU95 |
| Ascomycota | Leotiomycetes | Helotiales | Myxotrichaceae | Oidiodendron | *Oidiodendron periconioides* | OTU39 |
| Ascomycota | Leotiomycetes | Helotiales | Myxotrichaceae | Oidiodendron |  | OTU7 |
| Ascomycota | Leotiomycetes | Helotiales | Myxotrichaceae | Oidiodendron |  | OTU13 |
| Ascomycota | Leotiomycetes | Helotiales | Myxotrichaceae | Oidiodendron |  | OTU18 |
| Ascomycota | Leotiomycetes | Helotiales | Myxotrichaceae | Oidiodendron |  | OTU19 |
| Ascomycota | Leotiomycetes | Helotiales | Myxotrichaceae | Oidiodendron |  | OTU29 |
| Ascomycota | Leotiomycetes | Helotiales | Myxotrichaceae | Oidiodendron |  | OTU30 |
| Ascomycota | Leotiomycetes | Helotiales | Myxotrichaceae | Oidiodendron |  | OTU47 |
| Ascomycota | Leotiomycetes | Helotiales | Myxotrichaceae | Oidiodendron |  | OTU54 |
| Ascomycota | Leotiomycetes | Helotiales | Myxotrichaceae | Oidiodendron |  | OTU55 |
| Ascomycota | Leotiomycetes | Helotiales | Myxotrichaceae | Oidiodendron |  | OTU64 |
| Ascomycota | Leotiomycetes | Helotiales | Myxotrichaceae | Oidiodendron |  | OTU71 |
| Ascomycota | Leotiomycetes | Helotiales | Myxotrichaceae | Oidiodendron |  | OTU77 |
| Ascomycota | Leotiomycetes | Helotiales | Myxotrichaceae | Oidiodendron |  | OTU78 |
| Ascomycota | Leotiomycetes | Helotiales | Myxotrichaceae | Oidiodendron |  | OTU111 |
| Ascomycota | Leotiomycetes | Helotiales | Myxotrichaceae | Oidiodendron |  | OTU156 |
| Ascomycota | Leotiomycetes | Helotiales | Tympanidaceae |  |  | OTU184 |
| Ascomycota | Leotiomycetes | Helotiales |  |  |  | OTU17 |
| Ascomycota | Leotiomycetes | Helotiales |  |  |  | OTU23 |
| Ascomycota | Leotiomycetes | Helotiales |  |  |  | OTU24 |
| Ascomycota | Leotiomycetes | Helotiales |  |  |  | OTU33 |
| Ascomycota | Leotiomycetes | Helotiales |  |  |  | OTU59 |
| Ascomycota | Leotiomycetes | Helotiales |  |  |  | OTU70 |
| Ascomycota | Leotiomycetes | Helotiales |  |  |  | OTU73 |
| Ascomycota | Leotiomycetes | Helotiales |  |  |  | OTU90 |
| Ascomycota | Leotiomycetes | Helotiales |  |  |  | OTU101 |
| Ascomycota | Leotiomycetes | Helotiales |  |  |  | OTU138 |
| Ascomycota | Leotiomycetes | Helotiales |  |  |  | OTU139 |
| Ascomycota | Leotiomycetes | Helotiales |  |  |  | OTU141 |
| Ascomycota | Leotiomycetes | Helotiales |  |  |  | OTU145 |
| Ascomycota | Leotiomycetes | Helotiales |  |  |  | OTU149 |
| Ascomycota | Leotiomycetes | Helotiales |  |  |  | OTU161 |
| Ascomycota | Leotiomycetes | Helotiales |  |  |  | OTU165 |
| Ascomycota | Leotiomycetes | Helotiales |  |  |  | OTU198 |
| Ascomycota | Leotiomycetes | Rhytismatales | Rhytismataceae | Lophodermella | *Lophodermella conjuncta* | OTU62 |
| Ascomycota | Leotiomycetes | Rhytismatales | Rhytismataceae | Lophodermium | *Lophodermium pinastri* | OTU167 |
| Ascomycota | Leotiomycetes | Rhytismatales | Rhytismataceae |  |  | OTU89 |
| Ascomycota | Leotiomycetes | Thelebolales | Pseudeurotiaceae | Pseudogymnoascus |  | OTU57 |
| Ascomycota | Leotiomycetes |  |  |  |  | OTU43 |
| Ascomycota | Pezizomycetes | Pezizales | Pyronemataceae | Trichophaea |  | OTU4 |
| Ascomycota | Pezizomycetes | Pezizales | Pyronemataceae |  |  | OTU136 |
| Ascomycota | Saccharomycetes | Saccharomycetales | Debaryomycetaceae | Babjeviella | *Babjeviella inositovora* | OTU45 |
| Ascomycota | Saccharomycetes | Saccharomycetales | Saccharomycetaceae | Saccharomyces |  | OTU2 |
| Ascomycota | Saccharomycetes | Saccharomycetales | Trichomonascaceae | Sugiyamaella | *Sugiyamaella paludigena* | OTU88 |
| Ascomycota | Saccharomycetes | Saccharomycetales | Trichomonascaceae |  |  | OTU82 |
| Ascomycota | Sordariomycetes | Chaetosphaeriales | Chaetosphaeriaceae |  |  | OTU37 |
| Ascomycota | Sordariomycetes | Hypocreales | Clavicipitaceae | Pochonia |  | OTU32 |
| Ascomycota | Sordariomycetes | Hypocreales | Cordycipitaceae | Lecanicillium | *Lecanicillium flavidum* | OTU56 |
| Ascomycota | Sordariomycetes | Hypocreales | Hypocreaceae | Trichoderma | *Trichoderma viride* | OTU3 |
| Ascomycota | Sordariomycetes | Hypocreales | Hypocreaceae | Trichoderma | *Unclassified.Trichoderma* | OTU21 |
| Ascomycota | Sordariomycetes | Hypocreales | Ophiocordycipitaceae | Tolypocladium | *Tolypocladium album* | OTU85 |
| Ascomycota | Sordariomycetes | Xylariales | Hypoxylaceae | Hypoxylon | *Hypoxylon fendleri* | OTU144 |
| Ascomycota |  |  |  |  |  | OTU50 |
| Ascomycota |  |  |  |  |  | OTU130 |
| Ascomycota |  |  |  |  |  | OTU142 |
| Basidiomycota | Agaricomycetes | Agaricales | Cortinariaceae | Cortinarius | *Cortinarius neofurvolaesus* | OTU154 |
| Basidiomycota | Agaricomycetes | Agaricales | Cortinariaceae | Cortinarius |  | OTU155 |
| Basidiomycota | Agaricomycetes | Agaricales | Lycoperdaceae | Lycoperdon | *Lycoperdon pyriforme* | OTU5 |
| Basidiomycota | Agaricomycetes | Agaricales | Lyophyllaceae | Lyophyllum | *Lyophyllum shimeji* | OTU120 |
| Basidiomycota | Agaricomycetes | Atheliales | Atheliaceae | Athelia |  | OTU34 |
| Basidiomycota | Agaricomycetes | Atheliales | Pilodermataceae | Piloderma |  | OTU38 |
| Basidiomycota | Agaricomycetes | Atheliales | Pilodermataceae | Piloderma |  | OTU137 |
| Basidiomycota | Agaricomycetes | Atheliales | Pilodermataceae | Piloderma |  | OTU151 |
| Basidiomycota | Agaricomycetes | Atheliales | Tylosporaceae | Amphinema |  | OTU182 |
| Basidiomycota | Agaricomycetes | Atheliales | Tylosporaceae | Tylospora | *Tylospora asterophora* | OTU12 |
| Basidiomycota | Agaricomycetes | Cantharellales |  |  |  | OTU160 |
| Basidiomycota | Agaricomycetes | Polyporales | Meruliaceae | Ceraceomyces |  | OTU174 |
| Basidiomycota | Agaricomycetes | Russulales | Russulaceae | Lactarius | *Lactarius rufus* | OTU94 |
| Basidiomycota | Agaricomycetes | Sebacinales | Serendipitaceae | Serendipita |  | OTU135 |
| Basidiomycota | Agaricomycetes | Sebacinales | Serendipitaceae |  |  | OTU121 |
| Basidiomycota | Agaricomycetes | Thelephorales | Thelephoraceae | Thelephora | *Thelephora terrestris* | OTU53 |
| Basidiomycota | Agaricomycetes | Thelephorales | Thelephoraceae | Thelephora |  | OTU171 |
| Basidiomycota | Agaricomycetes | Thelephorales | Thelephoraceae | Tomentellopsis | *Tomentellopsis echinospora* | OTU191 |
| Basidiomycota | Agaricomycetes | Trechisporales | Hydnodontaceae | Trechispora |  | OTU164 |
| Basidiomycota | Agaricomycetes |  |  |  |  | OTU81 |
| Basidiomycota | Agaricomycetes |  |  |  |  | OTU124 |
| Basidiomycota | Geminibasidiomycetes | Geminibasidiales | Geminibasidiaceae | Geminibasidium |  | OTU143 |
| Basidiomycota | Microbotryomycetes | Kriegeriales | Kriegeriaceae | Yamadamyces |  | OTU185 |
| Basidiomycota | Microbotryomycetes | Leucosporidiales | Leucosporidiaceae | Leucosporidium |  | OTU148 |
| Basidiomycota | Tremellomycetes | Filobasidiales | Piskurozymaceae | Piskurozyma |  | OTU27 |
| Basidiomycota | Tremellomycetes | Filobasidiales | Piskurozymaceae | Solicoccozyma | *Solicoccozyma terricola* | OTU125 |
| Basidiomycota | Tremellomycetes | Tremellales | Phaeotremellaceae | Phaeotremella |  | OTU183 |
| Basidiomycota | Tremellomycetes | Tremellales | Syzygosporaceae | Syzygospora |  | OTU63 |
| Basidiomycota | Tremellomycetes | Tremellales | Tremellaceae | Tremella |  | OTU117 |
| Basidiomycota | Tremellomycetes | Tremellales | Trimorphomycetaceae | Saitozyma |  | OTU41 |
| Basidiomycota | Tremellomycetes | Tremellales |  |  |  | OTU35 |
| Basidiomycota | Tremellomycetes | Trichosporonales | Trichosporonaceae | Apiotrichum |  | OTU15 |
| Mortierellomycota | Mortierellomycetes | Mortierellales | Mortierellaceae | Mortierella | *Mortierella alliacea* | OTU28 |
| Mortierellomycota | Mortierellomycetes | Mortierellales | Mortierellaceae | Mortierella | *Mortierella alliacea* | OTU128 |
| Mortierellomycota | Mortierellomycetes | Mortierellales | Mortierellaceae | Mortierella | *Mortierella basiparvispora* | OTU80 |
| Mortierellomycota | Mortierellomycetes | Mortierellales | Mortierellaceae | Mortierella | *Mortierella macrocystis* | OTU26 |
| Mortierellomycota | Mortierellomycetes | Mortierellales | Mortierellaceae | Mortierella |  | OTU20 |
| Mortierellomycota | Mortierellomycetes | Mortierellales | Mortierellaceae | Mortierella |  | OTU22 |
| Mortierellomycota | Mortierellomycetes | Mortierellales | Mortierellaceae | Mortierella |  | OTU206 |
| Mortierellomycota | Mortierellomycetes | Mortierellales | Mortierellaceae |  |  | OTU163 |
| Mortierellomycota | Mortierellomycetes | Mortierellales | Mortierellaceae |  |  | OTU178 |
| Mucoromycota | Mucoromycetes | Mucorales | Mucoraceae | Mucor | *Mucor silvaticus* | OTU6 |
| Mucoromycota | Mucoromycetes | Mucorales | Mucoraceae | Mucor |  | OTU106 |
| Mucoromycota | Mucoromycetes | Mucorales | Mucoraceae | Mucor |  | OTU113 |
| Mucoromycota | Umbelopsidomycetes | Umbelopsidales | Umbelopsidaceae | Umbelopsis | *Umbelopsis ramanniana* | OTU72 |
| Mucoromycota | Umbelopsidomycetes | Umbelopsidales | Umbelopsidaceae | Umbelopsis |  | OTU31 |
| Mucoromycota | Umbelopsidomycetes | Umbelopsidales | Umbelopsidaceae | Umbelopsis |  | OTU60 |
| Mucoromycota | Umbelopsidomycetes | Umbelopsidales | Umbelopsidaceae | Umbelopsis |  | OTU83 |
| Mucoromycota | Umbelopsidomycetes | Umbelopsidales | Umbelopsidaceae | Umbelopsis |  | OTU104 |
| Mucoromycota | Umbelopsidomycetes | Umbelopsidales | Umbelopsidaceae | Umbelopsis |  | OTU108 |
| Mucoromycota |  |  |  |  |  | OTU58 |
| Rozellomycota |  |  |  |  |  | OTU40 |
| Unclassified Fungi |  |  |  |  |  | OTU9 |
| Unclassified Fungi |  |  |  |  |  | OTU25 |
| Unclassified Fungi |  |  |  |  |  | OTU91 |
| Unclassified Fungi |  |  |  |  |  | OTU195 |

**Table S6**. ASVs (181) occurring in all three treatments (MQ, sucrose, MQ plus litter, sucrose plus litter). The genus complex Burkholderia-Caballeronia-Paraburkholderia has been abbreviated to B.-C.-P.

| Phylum | Class | Order | Family | Genus | Species | ASV |
| --- | --- | --- | --- | --- | --- | --- |
| Acidobacteriota | Acidobacteriae | Acidobacteriales | Acidobacteriaceae (Subgroup 1) | Granulicella |  | ASV_33 |
| Acidobacteriota | Acidobacteriae | Acidobacteriales | Acidobacteriaceae (Subgroup 1) | Granulicella |  | ASV_44 |
| Acidobacteriota | Acidobacteriae | Acidobacteriales | Acidobacteriaceae (Subgroup 1) | Granulicella |  | ASV_68 |
| Acidobacteriota | Acidobacteriae | Acidobacteriales | Acidobacteriaceae (Subgroup 1) | Acidipila-Silvibacterium |  | ASV_71 |
| Acidobacteriota | Acidobacteriae | Acidobacteriales | Acidobacteriaceae (Subgroup 1) | Granulicella |  | ASV_78 |
| Acidobacteriota | Acidobacteriae | Acidobacteriales | Acidobacteriaceae (Subgroup 1) | Granulicella |  | ASV_87 |
| Acidobacteriota | Acidobacteriae | Acidobacteriales | Acidobacteriaceae (Subgroup 1) | Granulicella |  | ASV_90 |
| Acidobacteriota | Acidobacteriae | Acidobacteriales | Acidobacteriaceae (Subgroup 1) | Granulicella |  | ASV_94 |
| Acidobacteriota | Acidobacteriae | Acidobacteriales | Acidobacteriaceae (Subgroup 1) |  |  | ASV_97 |
| Acidobacteriota | Acidobacteriae | Acidobacteriales | Acidobacteriaceae (Subgroup 1) | Granulicella |  | ASV_103 |
| Acidobacteriota | Acidobacteriae | Acidobacteriales | Acidobacteriaceae (Subgroup 1) | Granulicella |  | ASV_109 |
| Acidobacteriota | Acidobacteriae | Acidobacteriales | Acidobacteriaceae (Subgroup 1) | Occallatibacter |  | ASV_111 |
| Acidobacteriota | Acidobacteriae | Acidobacteriales | Acidobacteriaceae (Subgroup 1) | Occallatibacter |  | ASV_116 |
| Acidobacteriota | Acidobacteriae | Acidobacteriales | Acidobacteriaceae (Subgroup 1) | Occallatibacter |  | ASV_125 |
| Acidobacteriota | Acidobacteriae | Acidobacteriales | Acidobacteriaceae (Subgroup 1) | Granulicella |  | ASV_127 |
| Acidobacteriota | Acidobacteriae | Acidobacteriales | Acidobacteriaceae (Subgroup 1) | Granulicella |  | ASV_140 |
| Acidobacteriota | Acidobacteriae | Acidobacteriales | Acidobacteriaceae (Subgroup 1) | Occallatibacter |  | ASV_144 |
| Acidobacteriota | Acidobacteriae | Acidobacteriales | Acidobacteriaceae (Subgroup 1) | Granulicella |  | ASV_150 |
| Acidobacteriota | Acidobacteriae | Acidobacteriales | Acidobacteriaceae (Subgroup 1) | Occallatibacter |  | ASV_155 |
| Acidobacteriota | Acidobacteriae | Acidobacteriales | Acidobacteriaceae (Subgroup 1) | Granulicella |  | ASV_160 |
| Acidobacteriota | Acidobacteriae | Acidobacteriales |  |  |  | ASV_165 |
| Acidobacteriota | Acidobacteriae | Acidobacteriales | Acidobacteriaceae (Subgroup 1) | Granulicella | *Granulicella mallensis* | ASV_170 |
| Acidobacteriota | Acidobacteriae | Acidobacteriales | Acidobacteriaceae (Subgroup 1) | Granulicella |  | ASV_174 |
| Acidobacteriota | Acidobacteriae | Acidobacteriales | Acidobacteriaceae (Subgroup 1) | Acidipila-Silvibacterium |  | ASV_206 |
| Acidobacteriota | Acidobacteriae | Acidobacteriales | Acidobacteriaceae (Subgroup 1) | Edaphobacter |  | ASV_211 |
| Acidobacteriota | Acidobacteriae | Acidobacteriales | Acidobacteriaceae (Subgroup 1) | Acidicapsa |  | ASV_224 |
| Acidobacteriota | Acidobacteriae | Acidobacteriales | Acidobacteriaceae (Subgroup 1) | Granulicella |  | ASV_239 |
| Acidobacteriota | Acidobacteriae | Acidobacteriales |  |  |  | ASV_243 |
| Acidobacteriota | Acidobacteriae | Acidobacteriales | Acidobacteriaceae (Subgroup 1) | Granulicella |  | ASV_250 |
| Acidobacteriota | Acidobacteriae | Acidobacteriales | Acidobacteriaceae (Subgroup 1) | Granulicella |  | ASV_252 |
| Acidobacteriota | Acidobacteriae | Acidobacteriales | Acidobacteriaceae (Subgroup 1) | Granulicella |  | ASV_277 |
| Acidobacteriota | Acidobacteriae | Acidobacteriales | Acidobacteriaceae (Subgroup 1) | Edaphobacter |  | ASV_285 |
| Acidobacteriota | Acidobacteriae | Subgroup 2 |  |  |  | ASV_293 |
| Acidobacteriota | Acidobacteriae | Acidobacteriales | Acidobacteriaceae (Subgroup 1) |  |  | ASV_337 |
| Actinobacteriota | Acidimicrobiia | |  |  |  | ASV_24 |
| Actinobacteriota | Acidimicrobiia | |  |  |  | ASV_29 |
| Actinobacteriota | Thermoleophilia | Solirubrobacterales | Solirubrobacteraceae | Conexibacter |  | ASV_38 |
| Actinobacteriota | Thermoleophilia | Solirubrobacterales | Solirubrobacteraceae | Conexibacter |  | ASV_39 |
| Actinobacteriota | Thermoleophilia | Solirubrobacterales | Solirubrobacteraceae |  |  | ASV_43 |
| Actinobacteriota | Thermoleophilia | Solirubrobacterales | Solirubrobacteraceae | Conexibacter |  | ASV_47 |
| Actinobacteriota | Actinobacteria | Corynebacteriales | Mycobacteriaceae | Mycobacterium |  | ASV_53 |
| Actinobacteriota | Actinobacteria | Corynebacteriales | Mycobacteriaceae | Mycobacterium |  | ASV_54 |
| Actinobacteriota | Thermoleophilia | Solirubrobacterales | Solirubrobacteraceae |  |  | ASV_69 |
| Actinobacteriota | Actinobacteria | Streptomycetales | Streptomycetaceae | Streptacidiphilus |  | ASV_70 |
| Actinobacteriota | Actinobacteria | Corynebacteriales | Mycobacteriaceae | Mycobacterium |  | ASV_72 |
| Actinobacteriota | Acidimicrobiia | |  |  |  | ASV_75 |
| Actinobacteriota | Thermoleophilia | Solirubrobacterales | Solirubrobacteraceae | Conexibacter |  | ASV_77 |
| Actinobacteriota | Thermoleophilia | Solirubrobacterales | Solirubrobacteraceae |  |  | ASV_79 |
| Actinobacteriota | Actinobacteria | Catenulisporales | Actinospicaceae | Actinospica |  | ASV_84 |
| Actinobacteriota | Acidimicrobiia | |  |  |  | ASV_88 |
| Actinobacteriota | Actinobacteria | Corynebacteriales | Mycobacteriaceae | Mycobacterium |  | ASV_100 |
| Actinobacteriota | Acidimicrobiia | IMCC26256 |  |  |  | ASV_102 |
| Actinobacteriota | Actinobacteria | Corynebacteriales | Mycobacteriaceae | Mycobacterium |  | ASV_119 |
| Actinobacteriota | Thermoleophilia | Solirubrobacterales | Solirubrobacteraceae | Conexibacter | *Conexibacter woesei* | ASV_133 |
| Actinobacteriota | Acidimicrobiia | IMCC26256 |  |  |  | ASV_135 |
| Actinobacteriota | Actinobacteria | Corynebacteriales | Mycobacteriaceae | Mycobacterium |  | ASV_137 |
| Actinobacteriota | Actinobacteria | Frankiales | Acidothermaceae | Acidothermus |  | ASV_148 |
| Actinobacteriota | Thermoleophilia | Solirubrobacterales | Solirubrobacteraceae | Conexibacter |  | ASV_157 |
| Actinobacteriota | Actinobacteria | Frankiales | Acidothermaceae | Acidothermus |  | ASV_161 |
| Actinobacteriota | Thermoleophilia | Solirubrobacterales | Solirubrobacteraceae | Conexibacter |  | ASV_175 |
| Actinobacteriota | Actinobacteria | Frankiales | Acidothermaceae | Acidothermus |  | ASV_178 |
| Actinobacteriota | Actinobacteria | Corynebacteriales | Mycobacteriaceae | Mycobacterium |  | ASV_185 |
| Actinobacteriota | Actinobacteria | Frankiales | Acidothermaceae | Acidothermus |  | ASV_195 |
| Actinobacteriota | Thermoleophilia | Solirubrobacterales | Solirubrobacteraceae | Conexibacter |  | ASV_199 |
| Actinobacteriota | Actinobacteria | Frankiales | Acidothermaceae | Acidothermus |  | ASV_203 |
| Actinobacteriota | Thermoleophilia | Solirubrobacterales | Solirubrobacteraceae | Conexibacter |  | ASV_204 |
| Actinobacteriota | Actinobacteria | Frankiales | Acidothermaceae | Acidothermus |  | ASV_208 |
| Actinobacteriota | Acidimicrobiia | |  |  |  | ASV_216 |
| Actinobacteriota | Thermoleophilia | Solirubrobacterales | Solirubrobacteraceae | Conexibacter |  | ASV_232 |
| Actinobacteriota | Actinobacteria | Corynebacteriales | Mycobacteriaceae | Mycobacterium | *Mycobacterium fortuitum* | ASV_233 |
| Actinobacteriota | Thermoleophilia | Solirubrobacterales | Solirubrobacteraceae | Conexibacter |  | ASV_235 |
| Actinobacteriota | Thermoleophilia | Solirubrobacterales | Solirubrobacteraceae |  |  | ASV_259 |
| Actinobacteriota | Actinobacteria | Frankiales | Acidothermaceae | Acidothermus |  | ASV_296 |
| Armatimonadota | Armatimonadia | Armatimonadales |  |  |  | ASV_57 |
| Armatimonadota | Armatimonadia | Armatimonadales |  |  |  | ASV_129 |
| Bacteroidota | Bacteroidia | Sphingobacteriales | CWT CU03-E12 |  |  | ASV_130 |
| Bacteroidota | Bacteroidia | Chitinophagales | Chitinophagaceae | Ferruginibacter |  | ASV_213 |
| Bacteroidota | Bacteroidia | Chitinophagales | Chitinophagaceae | Puia |  | ASV_247 |
| Bacteroidota | Bacteroidia | Chitinophagales | Chitinophagaceae | Puia |  | ASV_326 |
| Bdellovibrionota | Oligoflexia | 0319-6G20 |  |  |  | ASV_106 |
| Bdellovibrionota | Oligoflexia | 0319-6G20 |  |  |  | ASV_123 |
| Bdellovibrionota | Oligoflexia | 0319-6G20 |  |  |  | ASV_124 |
| Bdellovibrionota | Oligoflexia | 0319-6G20 |  |  |  | ASV_158 |
| Bdellovibrionota | Oligoflexia | 0319-6G20 |  |  |  | ASV_164 |
| Bdellovibrionota | Oligoflexia | 0319-6G20 |  |  |  | ASV_212 |
| Bdellovibrionota | Oligoflexia | 0319-6G20 |  |  |  | ASV_221 |
| Bdellovibrionota | Oligoflexia | 0319-6G20 |  |  |  | ASV_262 |
| Dependentiae | Babeliae | Babeliales | Vermiphilaceae |  |  | ASV_151 |
| Dependentiae | Babeliae | Babeliales |  |  |  | ASV_351 |
| Elusimicrobiota | Elusimicrobia | Lineage IV |  |  |  | ASV_368 |
| Proteobacteria | Gammaproteobacteria | Burkholderiales | Burkholderiaceae | Pandoraea |  | ASV_1 |
| Proteobacteria | Gammaproteobacteria | Burkholderiales | Oxalobacteraceae | Undibacterium |  | ASV_2 |
| Proteobacteria | Gammaproteobacteria | Burkholderiales | Oxalobacteraceae |  |  | ASV_3 |
| Proteobacteria | Gammaproteobacteria | Burkholderiales | Burkholderiaceae | Pandoraea |  | ASV_4 |
| Proteobacteria | Gammaproteobacteria | Burkholderiales | Burkholderiaceae | B.-C.-P. |  | ASV_5 |
| Proteobacteria | Gammaproteobacteria | Burkholderiales | Burkholderiaceae | B.-C.-P. |  | ASV_6 |
| Proteobacteria | Gammaproteobacteria | Burkholderiales | Burkholderiaceae | Pandoraea |  | ASV_7 |
| Proteobacteria | Gammaproteobacteria | Burkholderiales | Oxalobacteraceae |  |  | ASV_8 |
| Proteobacteria | Gammaproteobacteria | Burkholderiales | Burkholderiaceae | B.-C.-P. | *B.-C.-P.phenazinium* | ASV_9 |
| Proteobacteria | Gammaproteobacteria | Burkholderiales | Burkholderiaceae |  |  | ASV_10 |
| Proteobacteria | Gammaproteobacteria | Burkholderiales | Burkholderiaceae | B.-C.-P. | *B.-C.-P.caledonica* | ASV_11 |
| Proteobacteria | Gammaproteobacteria | Burkholderiales | Burkholderiaceae | B.-C.-P. | *B.-C.-P.phenazinium* | ASV_12 |
| Proteobacteria | Alphaproteobacteria | Rhodospirillales | Magnetospirillaceae |  |  | ASV_14 |
| Proteobacteria | Gammaproteobacteria | Burkholderiales | Burkholderiaceae | B.-C.-P. |  | ASV_16 |
| Proteobacteria | Gammaproteobacteria | Burkholderiales | Burkholderiaceae | Ralstonia | *Ralstonia solanacearum* | ASV_18 |
| Proteobacteria | Gammaproteobacteria | Xanthomonadales | Rhodanobacteraceae | Rhodanobacter |  | ASV_21 |
| Proteobacteria | Alphaproteobacteria | Rhizobiales | Xanthobacteraceae | Bradyrhizobium | *Bradyrhizobium elkanii* | ASV_22 |
| Proteobacteria | Gammaproteobacteria | Burkholderiales | Burkholderiaceae | B.-C.-P. | *B.-C.-P.phenazinium* | ASV_25 |
| Proteobacteria | Alphaproteobacteria | Rhizobiales | Xanthobacteraceae | Bradyrhizobium | *Bradyrhizobium elkanii* | ASV_26 |
| Proteobacteria | Gammaproteobacteria | Burkholderiales | Oxalobacteraceae | Undibacterium |  | ASV_27 |
| Proteobacteria | Gammaproteobacteria | Burkholderiales | Burkholderiaceae | Pandoraea |  | ASV_28 |
| Proteobacteria | Gammaproteobacteria | Burkholderiales | Oxalobacteraceae | Undibacterium |  | ASV_31 |
| Proteobacteria | Gammaproteobacteria | Burkholderiales | Burkholderiaceae | B.-C.-P. | *B.-C.-P.terricola* | ASV_34 |
| Proteobacteria | Gammaproteobacteria | Burkholderiales | Burkholderiaceae | B.-C.-P. | *B.-C.-P.phenazinium* | ASV_35 |
| Proteobacteria | Alphaproteobacteria | Rhizobiales | Xanthobacteraceae | Bradyrhizobium | *Bradyrhizobium canariense* | ASV_36 |
| Proteobacteria | Gammaproteobacteria | Burkholderiales | Burkholderiaceae | B.-C.-P. | *B.-C.-P.phenazinium* | ASV_37 |
| Proteobacteria | Gammaproteobacteria | Xanthomonadales | Rhodanobacteraceae | Dyella |  | ASV_41 |
| Proteobacteria | Gammaproteobacteria | Burkholderiales | Burkholderiaceae | B.-C.-P. |  | ASV_46 |
| Proteobacteria | Gammaproteobacteria | Burkholderiales | Burkholderiaceae | B.-C.-P. | *B.-C.-P.phenazinium* | ASV_48 |
| Proteobacteria | Gammaproteobacteria | Salinisphaerales | Solimonadaceae | Nevskia | *Nevskia soli* | ASV_49 |
| Proteobacteria | Gammaproteobacteria | Burkholderiales | Burkholderiaceae | B.-C.-P. | *B.-C.-P.phenazinium* | ASV_50 |
| Proteobacteria | Alphaproteobacteria | Rhizobiales | Beijerinckiaceae | Roseiarcus |  | ASV_51 |
| Proteobacteria | Alphaproteobacteria | Micropepsales | Micropepsaceae |  |  | ASV_52 |
| Proteobacteria | Gammaproteobacteria | WD260 |  |  |  | ASV_55 |
| Proteobacteria | Alphaproteobacteria | Sphingomonadales | Sphingomonadaceae | Novosphingobium | *Novosphingobium acidiphilum* | ASV_58 |
| Proteobacteria | Alphaproteobacteria | Acetobacterales | Acetobacteraceae | Acidocella |  | ASV_59 |
| Proteobacteria | Gammaproteobacteria | WD260 |  |  |  | ASV_60 |
| Proteobacteria | Alphaproteobacteria | Caulobacterales | Caulobacteraceae | Phenylobacterium |  | ASV_61 |
| Proteobacteria | Gammaproteobacteria | WD260 |  |  |  | ASV_63 |
| Proteobacteria | Alphaproteobacteria | Rhizobiales | Beijerinckiaceae | Roseiarcus |  | ASV_66 |
| Proteobacteria | Gammaproteobacteria | Burkholderiales | Burkholderiaceae |  |  | ASV_67 |
| Proteobacteria | Alphaproteobacteria | Rhizobiales | Xanthobacteraceae | Bradyrhizobium | *Bradyrhizobium erythrophlei* | ASV_73 |
| Proteobacteria | Alphaproteobacteria | Rhizobiales | Beijerinckiaceae |  |  | ASV_74 |
| Proteobacteria | Gammaproteobacteria | Burkholderiales | Burkholderiaceae | B.-C.-P. | *B.-C.-P.bryophila* | ASV_76 |
| Proteobacteria | Gammaproteobacteria | |  |  |  | ASV_80 |
| Proteobacteria | Alphaproteobacteria | Rhizobiales | Rhizobiaceae | Mesorhizobium | *Mesorhizobium plurifarium* | ASV_81 |
| Proteobacteria | Gammaproteobacteria | Burkholderiales | Burkholderiaceae | B.-C.-P. | *B.-C.-P.phytofirmans* | ASV_82 |
| Proteobacteria | Gammaproteobacteria | WD260 |  |  |  | ASV_93 |
| Proteobacteria | Alphaproteobacteria | Caulobacterales | Caulobacteraceae | Phenylobacterium |  | ASV_95 |
| Proteobacteria | Gammaproteobacteria | Gammaproteobacteria | Unknown Family | Acidibacter |  | ASV_96 |
| Proteobacteria | Gammaproteobacteria | Salinisphaerales | Solimonadaceae | Nevskia |  | ASV_98 |
| Proteobacteria | Gammaproteobacteria | Burkholderiales | Burkholderiaceae | B.-C.-P. |  | ASV_99 |
| Proteobacteria | Alphaproteobacteria | Caulobacterales | Caulobacteraceae |  |  | ASV_101 |
| Proteobacteria | Alphaproteobacteria | Rhizobiales | Beijerinckiaceae | Roseiarcus |  | ASV_112 |
| Proteobacteria | Alphaproteobacteria | Acetobacterales | Acetobacteraceae | Rhodovastum |  | ASV_114 |
| Proteobacteria | Alphaproteobacteria | Rhodospirillales |  |  |  | ASV_115 |
| Proteobacteria | Gammaproteobacteria | Burkholderiales | Burkholderiaceae | B.-C.-P. |  | ASV_117 |
| Proteobacteria | Alphaproteobacteria | Acetobacterales | Acetobacteraceae | Acidocella |  | ASV_121 |
| Proteobacteria | Gammaproteobacteria | Gammaproteobacteria | Unknown Family | Acidibacter |  | ASV_126 |
| Proteobacteria | Alphaproteobacteria | Acetobacterales | Acetobacteraceae | Acidocella |  | ASV_128 |
| Proteobacteria | Alphaproteobacteria | Rhizobiales | Xanthobacteraceae |  |  | ASV_136 |
| Proteobacteria | Gammaproteobacteria | Xanthomonadales | Rhodanobacteraceae | Dyella |  | ASV_141 |
| Proteobacteria | Alphaproteobacteria | Acetobacterales | Acetobacteraceae | Acidocella |  | ASV_142 |
| Proteobacteria | Alphaproteobacteria | Rhizobiales | Beijerinckiaceae | Methylocella |  | ASV_159 |
| Proteobacteria | Alphaproteobacteria | Caulobacterales | Caulobacteraceae |  |  | ASV_163 |
| Proteobacteria | Gammaproteobacteria | |  |  |  | ASV_167 |
| Proteobacteria | Alphaproteobacteria | Rhizobiales | Beijerinckiaceae |  |  | ASV_169 |
| Proteobacteria | Gammaproteobacteria | |  |  |  | ASV_172 |
| Proteobacteria | Alphaproteobacteria | Azospirillales | Inquilinaceae | Inquilinus |  | ASV_177 |
| Proteobacteria | Alphaproteobacteria | Micropepsales | Micropepsaceae |  |  | ASV_180 |
| Proteobacteria | Gammaproteobacteria | Diplorickettsiales | Diplorickettsiaceae |  |  | ASV_181 |
| Proteobacteria | Gammaproteobacteria | Pseudomonadales | Pseudomonadaceae | Pseudomonas |  | ASV_187 |
| Proteobacteria | Alphaproteobacteria | Caulobacterales | Caulobacteraceae | Phenylobacterium |  | ASV_188 |
| Proteobacteria | Gammaproteobacteria | WD260 |  |  |  | ASV_194 |
| Proteobacteria | Alphaproteobacteria | Caulobacterales | Caulobacteraceae | Asticcacaulis |  | ASV_215 |
| Proteobacteria | Alphaproteobacteria | Acetobacterales | Acetobacteraceae |  |  | ASV_218 |
| Proteobacteria | Alphaproteobacteria | Rhizobiales | Beijerinckiaceae | Roseiarcus |  | ASV_219 |
| Proteobacteria | Alphaproteobacteria | Caulobacterales | Caulobacteraceae | Phenylobacterium |  | ASV_225 |
| Proteobacteria | Alphaproteobacteria | Rhizobiales | Beijerinckiaceae |  |  | ASV_244 |
| Proteobacteria | Alphaproteobacteria | Rhizobiales | Beijerinckiaceae |  |  | ASV_256 |
| Proteobacteria | Alphaproteobacteria | Acetobacterales | Acetobacteraceae |  |  | ASV_266 |
| Proteobacteria | Alphaproteobacteria | Acetobacterales | Acetobacteraceae | Acidisoma | *Acidisoma sibiricum* | ASV_269 |
| Proteobacteria | Alphaproteobacteria | Caulobacterales | Caulobacteraceae | Phenylobacterium |  | ASV_271 |
| Proteobacteria | Gammaproteobacteria | Burkholderiales | Burkholderiaceae | Cupriavidus | *Cupriavidus basilensis* | ASV_283 |
| Proteobacteria | Alphaproteobacteria | Rhodospirillales |  |  |  | ASV_294 |
| Proteobacteria | Gammaproteobacteria | |  |  |  | ASV_303 |
| Proteobacteria | Alphaproteobacteria | |  |  |  | ASV_310 |
| Verrucomicrobiota | Chlamydiae | Chlamydiales | Parachlamydiaceae | Neochlamydia |  | ASV_83 |
| Verrucomicrobiota | Chlamydiae | Chlamydiales |  |  |  | ASV_110 |
| Verrucomicrobiota | Chlamydiae | Chlamydiales | Parachlamydiaceae | Neochlamydia |  | ASV_230 |
| Verrucomicrobiota | Chlamydiae | Chlamydiales | Parachlamydiaceae | Candidatus Protochlamydia |  | ASV_267 |
